# Selective inhibition of TRPM2 channel by two novel synthesized ADPR analogues

**DOI:** 10.1111/cbdd.13119

**Published:** 2017-11-15

**Authors:** Xiao Luo, Meng Li, Kaiyu Zhan, Wei Yang, Lihe Zhang, KeWei Wang, Peilin Yu, Liangren Zhang

**Affiliations:** ^1^ State Key Laboratory of Natural and Biomimetic Drugs Peking University Beijing China; ^2^ Department of Neurobiology Neuroscience Research Institute Peking University Health Science Center Peking University School of Pharmaceutical Sciences Beijing China; ^3^ Department of Neurobiology Zhejiang University School of Medicine Hangzhou Zhejiang China; ^4^ Department of Pharmacology School of Pharmacy Qingdao University Qingdao China; ^5^ Department of Toxicology School of Public Health Zhejiang University Hangzhou Zhejiang China

**Keywords:** ADPR analogues, inhibitor, selectivity, TRP channels, TRPM2

## Abstract

Transient receptor potential melastatin‐2 (TRPM2) channel critical for monitoring internal body temperature is implicated in the pathological processes such as neurodegeneration. However, lacking selective and potent TRPM2 inhibitors impedes investigation and validation of the channel as a drug target. To discover novel and selective TRPM2 inhibitors, a series of adenosine 5′‐diphosphoribose analogues were synthesized, and their activities and selectivity were evaluated. Whole‐cell patch‐clamp recordings were employed for screen and evaluation of synthesized compounds. Two compounds, **7i** and **8a**, were identified as TRPM2 inhibitors with IC_50_ of 5.7 and 5.4 μm, respectively. Both **7i** and **8a** inhibited TRPM2 current without affecting TRPM7, TRPM8, TRPV1 and TRPV3. These two TRPM2 inhibitors can serve as new pharmacological tools for further investigation and validation of TRPM2 channel as a drug target, and the summarized structure–activity relationship (SAR) may also provide insights into further improving existing inhibitors as potential lead compounds.

## 1 INTRODUCTION

Transient receptor potential (TRP) channels are involved in diverse physiological functions including thermosensation and chemosensation.^1^, ^2^ The mammalian TRP superfamily is composed of 28 members that are grouped into six subfamilies based on their amino acid sequence homology: TRPC (canonical or classic), TRPV (vanilloid), TRPM (melastatin), TRPA (ankyrin), TRPP (polycystin) and TRPML (mucolipin).^3^ TRPM2, a subtype of TRP‐melastatin subfamily (TRPM), is a multifunctional Ca^2+^ permeable and nonselective cation channel.^4^ It functions as an important Ca^2+^ signalling regulator in a variety of cells, contributing to cellular functions including cytokine production, insulin release, cell motility, oxidative stress and cell death.^5^ TRPM2 is activated by reactive oxygen species (ROS) such as hydrogen peroxide and is considered as a chanzyme containing a unique C‐terminal Nudix hydrolase domain, that is homologous to the ADPR pyrophosphatase nudix hydrolase 9 (NUDT9).^6^, ^7^, ^8^ The NUDT9‐homology (NUDT9‐H) domain serves a binding site for channel activation by adenosine 5′‐diphosphoribose (ADPR).^6^ Accumulating evidence shows that TRPM2 channel plays a critical role in sensing internal body temperature^9^, ^10^, ^11^ and is also implicated in many pathological processes such as neurodegeneration,^12^, ^13^ diabetes^14^, ^15^ and ischemic reperfusion injury,^16^, ^17^ suggesting TRPM2 as a potential therapeutic target. Therefore, inhibiting TRPM2 may lead to a therapeutic potential for ischemicinjury.

**Figure 1:**
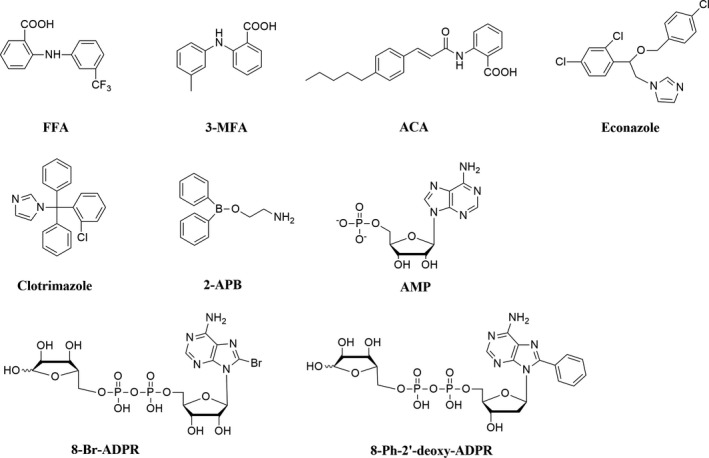
Structures of TRPM2 inhibitors from reported literatures

In the past, several small molecules (Figure 1) have been reported to show TRPM2 inhibitory activity with variable IC_50_ (1.2~76 μm), including flufenamic acid (FFA),^18^, ^19^ 2‐(3‐methylphenyl) aminobenzoic acid (3‐MFA),^19^ *N*‐(*p*‐amylcinnamoyl) anthranilic acid (ACA),^20^ econazole, clotrimazole^21^ and 2‐aminoethoxydiphenyl borate (2‐APB).^22^, ^23^ However, none of them show any specificity against TRPM2 channel.^24^ For instance, 2‐APB activates TRPV1, TRPV2 and TRPV3.^25^ In addition, several nucleotide analogues such as adenosine monophosphate (AMP), 8‐bromoadenosine 5′‐diphosphoribose (8‐Br‐ADPR) have also been reported to be TRPM2 inhibitors (Figure 1).^26^, ^27^ Recently, a series of ADPR analogues mainly modified at C‐8 position of purine such as 8‐phenyl‐2′‐*deoxy*‐ADPR (Figure 1) have been shown to potently inhibit TRPM2 current (IC_50_ = 3 μm) without interfering Ca^2+^ release induced by cADPR, NAADP or IP_3_.^28^ Despite the efforts in searching for TRPM2 inhibitors, no TRP‐subtype selective TRPM2 inhibitor has yet been identified. Therefore, it is critical to discover novel selective inhibitors of TRPM2 that can be used to further investigate TRPM2 channel function and validate the channel as a therapeutic target.

**Figure 2:**
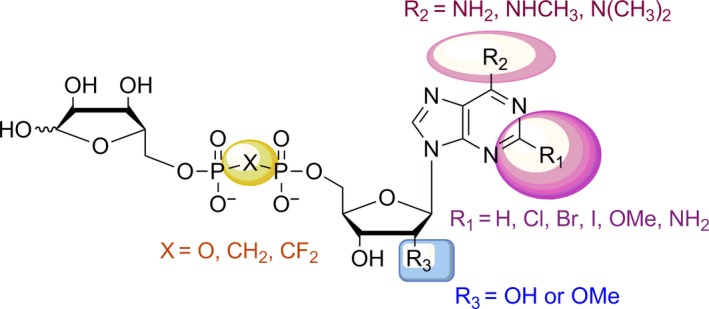
General structures of ADPR analogues [Colour figure can be viewed at wileyonlinelibrary.com]

Substitution of pyrophosphate linkage oxygen of ADPR has rarely been investigated. Xu et al.^29^ introduced a methylenebisphosphonate linkage to cADPR and found that the activity of cADPR is very sensitive to the modifications in the pyrophosphate moiety, indicating that pyrophosphate moiety contributes to cADPR binding on its receptor. Considering the homology of cADPR and ADPR, we, in this study, synthesized a new series of ADPR analogues (Figure 2) by integrating the modifications of pyrophosphate linkage and adenosine, in which the pyrophosphate linkage was replaced by methylenebisphosphate or difluoromethylenebisphosphate. The activity of all synthesized individual ADPR analogues was assessed. Their selectivity against other TRP channels including TRPM7, TRPM8, TRPV1 and TRPV3 was also evaluated. Two novel TRPM2 inhibitors, **7i** and **8a**, were identified in this study, and both compounds can be primarily used to probe physiological and pharmacological functions of TRPM2 channel, and their potency can also be further improved.

## 2 EXPERIMENTAL SECTION

### 2.1 Chemistry

All commercial chemicals and solvents were purchased from commercial suppliers of analytical grade and used without further purification. ^1^H and ^13^C NMR spectra were recorded on a Bruker Avance III 400 spectrometer. Chemical shifts were reported as values from an internal tetramethylsilane standard. High‐resolution mass spectra (HRMS) were recorded on a Bruker Apex IV FTMS. LC/MS analyses were performed on an Agilent 1200‐6110 instruments. Silica gel (200~300 mesh) was used for chromatography. HPLC (Gilson, France) was used to purify the protected and the final deprotected ADPR analogues, whereby a C18 reversed‐phase column (Venusil XBP C18‐2, 21.5 mm×250 mm, 10 μm, Agela Technologies, Beijing, China) was employed.

**Figure 3:**
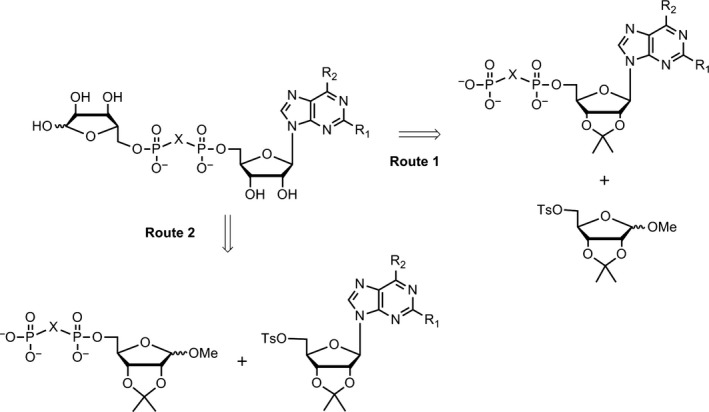
Routes for the synthesis of ADPR analogues

Generally, the synthesis of ADPR analogues could be divided into two routes (Figure 3). One was adenosine diphosphate (ADP) coupled with 5‐tosyl ribose (Figure 3), in which ADP analogues were originally synthesized from C‐2 position substituted adenosines and 5‐tosyl ribose was originally synthesized from D‐ribose. The other one was 5′‐tosyl nucleoside coupled with ribose diphosphate (RDP) (Figure 3), in which 5‐tosyl adenosines were originally synthesized from C‐2 position substituted adenosine and RDP analogues were originally synthesized from D‐ribose. Protected ADPR analogues were synthesized employing above synthetic route and were further deprotected with aqueous HCl and purified by HPLC to give the final ADPR analogues. Detailed characterization data by ^1^H, ^13^C NMR, MS and HRMS for all compounds are available in Supporting information.

#### 2.1.1 Synthetic route 1 and general procedures

2′,3′‐*O*‐Isopropylidene‐5′‐*O*‐tosyl adenosine **2a** was prepared with similar procedures according to the references.^30^, ^31^ Compound **2a** (0.65 g, 1.4 mmol) and tris(tetra‐*n*‐butylammonium) hydrogen methylenediphosphonate (1.60 g, 1.8 mmol) were mixed with 3 ml dry CH_3_CN and stirred at room temperature for 16 hr. The solvent was evaporated, diluted with H_2_O and eluted through a column of Amberlyst A15 resin (NH_4_^+^ form). The eluant was concentrated to dryness. The residue was separated on silica gel chromatography (iPrOH:H_2_O:aq NH_3_ = 8:1:1 to 6:2:2) to give the ammonium salt of 2′,3′‐*O*‐isopropylidene‐5′‐methylenediphosphate adenosine **3a** as a light yellow foam solid (0.52 g, yield: 67%). Compound **3a** (520 mg, 1.0 mmol) was treated with tetrabutylammonium hydroxide (40%wt in water, 1.94 ml, 3.0 mmol) and lyophilized. Then 1‐*O*‐methyl‐2,3‐*O*‐isopropylidene‐5‐*O*‐tosyl ribose **5** and 1 ml dry CH_3_CN were added and stirred at room temperature for 48 hr. The solvent was evaporated, diluted with H_2_O and eluted through a column of Amberlyst A15 resin (NH_4_^+^ form). The eluant was concentrated to dryness. The residue was separated on silica gel chromatography (iPrOH:H_2_O:aq NH_3_ = 16:1:1 to 8:1:1), followed by further purification with HPLC (H_2_O:CH_3_CN = 9:1 to 1:1) to give the ammonium salt of 1″‐*O*‐methyl‐2″,2′,3″,3′‐*O*‐isopropylidene‐5′‐methylenediphoribose adenosine **7a** (protected CH_2_ADPR) as a white foam solid (140 mg, yield: 20%). Compound **7a** (69 mg, 0.1 mmol) was treated with 0.8 N aqueous HCl at 0°C for 8 hr, purified with HPLC (50 mmol aq NH_4_HCO_3_:CH_3_CN = 20:1 to 1:2) to give 1″‐*O*‐methyl‐2″,3″‐*O*‐isopropylidene‐5′‐methylenediphosphoribose adenosine **8a** as a foam solid (21 mg, yield: 32%) and impure methylenediphosphoribose adenosine **10a**, which was further purified by HPLC (50 mmol aq triethylamine acetate:CH_3_CN = 20:1 to 4:1) to give **10a** (CH_2_ADPR) as a foam solid (16 mg, yield: 25%).

**7a** ^1^H NMR (400 MHz, D_2_O) δ 8.39 (s, 1H), 8.11 (s, 1H), 6.14 (d, *J *=* *3.3 Hz, 1H), 5.31 (dd, *J *=* *6.1, 3.3 Hz, 1H), 5.13 (dd, *J *=* *6.1, 2.1 Hz, 1H), 4.87 (s, 1H), 4.65 (d, *J *=* *6.0 Hz, 1H), 4.56 (d, *J *=* *1.8 Hz, 1H), 4.47 (d, *J *=* *5.9 Hz, 1H), 4.15 (t, *J *=* *7.2 Hz, 1H), 4.10–3.91 (m, 2H), 3.83–3.50 (m, 2H), 3.20 (s, 3H), 2.03 (t, *J *=* *19.1 Hz, 2H), 1.59 (s, 3H), 1.37 (s, 3H), 1.32 (s, 3H), 1.18 (s, 3H). ^31^P NMR (162 MHz, D_2_O) δ 17.0 (m). ^13^C NMR (101 MHz, D_2_O) δ 154.9, 152.0, 148.6, 140.4, 118.5, 115.0, 112.9, 108.5, 90.1, 85.1, 85.1, 85.1, 85.0, 84.1, 84.0, 81.4, 81.1, 64.2, 54.8, 26.2, 25.2, 24.5, 23.6. HRMS (ESI^+^): Calcd for C_23_H_36_N_5_O_13_P_2_ [*M* + H]^+^: 652.1785; Found: 652.1786.

**8a** ^1^H NMR (400 MHz, D_2_O) δ 8.48 (s, 1H), 8.14 (s, 1H), 6.01 (d, *J *=* *5.5 Hz, 1H), 4.85 (s, 1H), 4.66 (t, *J *=* *5.5 Hz, 1H), 4.60 (d, *J *=* *6.0 Hz, 1H), 4.45–4.41 (m, 2H), 4.26 (d, *J *=* *6.0 Hz, 1H), 4.17–4.02 (m, 3H), 3.68 (ddd, *J *=* *24.3, 14.1, 7.2 Hz, 2H), 3.18 (s, 3H), 2.09 (t, *J *=* *20.0 Hz, 2H), 1.24 (s, 3H), 1.11 (s, 3H). ^31^P NMR (162 MHz, D_2_O) δ 17.0 (m). ^13^C NMR (101 MHz, D_2_O) δ 156.3, 153.8, 150.2, 140.1, 117.7, 112.8, 108.5, 87.1, 85.0, 84.9, 84.1, 83.9, 81.0, 74.3, 70.2, 64.2, 63.6, 54.7, 25.1, 23.5. HRMS (ESI^+^): Calcd for C_20_H_32_N_5_O_13_P_2_ [*M* + H]^+^: 612.1472; Found: 612.1462.

**10a** ^1^H NMR (400 MHz, D_2_O) δ 8.49 (s, 1H), 8.18 (s, 1H), 6.06 (d, *J *=* *5.6 Hz, 1H), 5.20 (5.27, d, *J *=* *4.1 Hz, 0.4H, 5.14, d, *J *=* *2.1 Hz, 0.6H), 4.73 (s, 1H, partially hidden under HDO peak), 4.56–4.46 (m, 1H), 4.32 (d, *J *=* *3.1 Hz, 1H), 4.29–4.23 (m, 1H), 4.17–4.09 (m, 3H), 4.05–3.88 (m, 3H), 2.16 (ddd, *J *=* *27.5, 14.3, 5.0 Hz, 2H). ^31^P NMR (162 MHz, D_2_O) δ 18.1 (m). ^13^C NMR (101 MHz, D_2_O) δ 154.8, 151.8, 149.0, 140.4, 118.6, 101.3, 96.4, 87.1, 84.2, 82.0, 75.3, 74.3, 70.7, 70.4, 64. 6, 63.6. HRMS (ESI^+^): Calcd for C_16_H_26_N_5_O_13_P_2_ [*M* + H]^+^: 558.1001; Found: 558.1001.

#### 2.1.2 Synthetic route 2 and general procedures

Acetyl chloride (0.14 g, 1.8 mmol) was added dropwise to 30 ml CH_3_OH suspension of D‐ribose (3.5 g, 23.3 mmol) at 0°C, stirred at room temperature for additional 3 hr. The solution was neutralized with saturated NaHCO_3_, and the solvent was evaporated to give a light yellow syrup. Triethyl orthoformate (10.0 g, 68 mmol) and TsOH·H_2_O (0.75 g, 3.9 mmol) were stirred with 100 ml acetone at 50°C overnight, then this solution was added to above syrup and stirred overnight. The solvent was evaporated, and the residue was separated on silica gel chromatography (CH_2_Cl_2_:MeOH = 50:1) to give 1‐*O*‐methyl‐2, 3‐*O*‐isopropylidene ribose (1.67 g, 35%). Above product was stirred with TsCl (3.1 g, 16.3 mmol) and Et_3_N (4 ml, 31 mmol) for 3 hr. The solvent was evaporated, and the residue was separated on silica gel chromatography (petrol:EtOAc = 4:1) to give the 1‐*O*‐methyl‐2,3‐*O*‐isopropylidene‐5‐*O*‐tosyl ribose **5** (2.05 g, 70%). Compound **5** (413 mg, 1.0 mmol) and tris(tetra‐*n*‐butylammonium) hydrogen difluoromethylenediphosphonate (1.06 g, 1.2 mmol) were mixed with 3 ml dry CH_3_CN and stirred at room temperature for 24 hr. The solvent was evaporated, diluted with H_2_O and eluted through a column of Amberlyst A15 resin (NH_4_^+^ form). The eluant was concentrated to dryness. The residue was separated on silica gel chromatography (iPrOH:H_2_O:aq NH_3_ = 8:1:1 to 6:2:2) to give the ammonium salt of 1‐*O*‐methyl‐2,3‐*O*‐isopropylidene‐5‐difluoromethylenediphosphate ribose **6b** as a white foam solid (343 mg, yield: 70%). Compound **6b** (343 mg, 0.7 mmol) was treated with tetrabutylammonium hydroxide (40%wt in water, 1.36 ml, 2.1 mmol) and lyophilized. Then 2′,3′‐*O*‐isopropylidene‐5′‐*O*‐tosyl‐2‐bromoadenosine **2c** (264 mg, 0.5 mmol) and 1 ml dry CH_3_CN were added and stirred at room temperature for 36 hr. The solvent was evaporated, diluted with H_2_O and eluted through a column of Amberlyst A15 resin (NH_4_^+^ form). The eluant was concentrated to dryness. The residue was separated on silica gel chromatography (iPrOH:H_2_O:aq NH_3_ = 16:1:1 to 8:1:1) to give the ammonium salt of 1″‐*O*‐methyl‐2″,2′,3″,3′‐*O*‐isopropylidene‐5′‐difluoromethylenediphosphoribose‐2‐bromoadenosine **7n** (protected 2‐Br‐CF_2_ADPR) as a white foam solid (236 mg, yield: 50%). Compound **7n** (83 mg, 0.1 mmol) was treated with 0.8 N aqueous HCl at 0°C for 8 hr to give 1″‐*O*‐methyl‐2″,3″‐*O*‐isopropylidene‐5′‐difluoromethylenediphosphoribose‐2‐bromoadenosine **8d** as a foam solid (26 mg, yield: 33%), 1″‐*O*‐methyl‐difluoromethylenediphosphoribose‐2‐bromoadenosine **9a** as a foam solid (20 mg, yield: 28%) and difluoromethylenediphosphoribose‐2‐bromoadenosine **10n** (2‐Br‐CF_2_ADPR) as a foam solid (18 mg, yield: 24%).

**7n** ^1^H NMR (400 MHz, D_2_O) δ 8.29 (s, 1H), 6.08 (d, *J* = 3.3 Hz, 1H), 5.30 (dd, *J *=* *6.0, 3.4 Hz, 1H), 5.12 (dd, *J *=* *6.0, 2.1 Hz, 1H), 4.86 (s, 1H), 4.55 (dd, *J *=* *7.3, 4.1 Hz, 2H), 4.44 (d, *J *=* *5.9 Hz, 1H), 4.22–4.11 (m, 2H), 4.08 (dd, *J *=* *14.4, 7.1 Hz, 1H), 3.93–3.66 (m, 2H), 3.20 (s, 3H), 1.59 (s, 3H), 1.38 (s, 3H), 1.31 (s, 3H), 1.16 (s, 3H). ^19^F NMR (376 MHz, D_2_O) δ −118.7 (td, *J *=* *83.3, 35.4 Hz). ^31^P NMR (162 MHz, D_2_O) δ 4.1 (m). ^13^C NMR (101 MHz, D_2_O) δ 155.8, 149.7, 144.5, 140.0, 117.7, 115.0, 112.9, 108.5, 89.7, 85.0, 84.9, 84.9, 84.8, 84.1, 83.9, 81.3, 80.9, 66.4, 54.8, 26.2, 25.2, 24.4, 23.5. HRMS (ESI^+^): Calcd for C_23_H_33_F_2_N_5_O_13_P_2_Br [*M* + H]^+^: 766.0702; Found: 766.0696.

**8d** ^1^H NMR (400 MHz, D_2_O) δ 8.37 (s, 1H), 5.94 (d, *J *=* *5.6 Hz, 1H), 4.88 (s, 1H), 4.69–4.61 (t, *J *=* *5.2 Hz, 1H), 4.57 (d, *J *=* *6.0 Hz, 1H), 4.47 (d, *J *=* *5.9 Hz, 1H), 4.42 (dd, *J *=* *5.0, 3.8 Hz, 1H), 4.28 (d, *J *=* *1.8 Hz, 1H), 4.22 (d, *J *=* *4.5 Hz, 2H), 4.11 (t, *J *=* *7.3 Hz, 1H), 3.90–3.73 (m, 2H), 3.21 (s, 3H), 1.29 (s, 3H), 1.14 (s, 3H). ^19^F NMR (376 MHz, D_2_O) δ −118.6 (t, *J *=* *83.5 Hz). ^31^P NMR (162 MHz, D_2_O) δ 4.2 (m). ^13^C NMR (101 MHz, D_2_O) δ 156.1, 150.2, 144.5, 139.8, 118.0, 112.9, 108.5, 87.0, 84.8, 84.8, 84.2, 84.1, 80.9, 74.4, 70.4, 66.2, 65.6, 54.8, 25.1, 23.4. HRMS (ESI^+^): Calcd for C_20_H_29_F_2_N_5_O_13_P_2_Br [*M* + H]^+^: 726.0389; Found: 726.0404.

**9a** ^1^H NMR (400 MHz, D_2_O) δ 8.30 (s, 1H), 5.89 (d, *J *=* *5.4 Hz, 1H), 4.74 (1H, partially hidden under HDO peak), 4.58 (t, *J *=* *5.2 Hz, 1H), 4.43–4.34 (m, 1H), 4.24 (d, *J *=* *2.9 Hz, 1H), 4.18 (s, 2H), 4.11 (dd, *J *=* *6.1, 5.0 Hz, 1H), 4.07–3.87 (m, 4H), 3.23 (s, 3H).^19^F NMR (376 MHz, D_2_O) δ −118.7 (t, *J *=* *82.4 Hz). ^31^P NMR (162 MHz, D_2_O) δ 4.2 (m). ^13^C NMR (101 MHz, D_2_O) δ 155.9, 149.9, 144.4, 139.7, 117.9, 107.8, 90.2, 87.1, 83.9, 81.5, 74.4, 74.0, 70.7, 70.1, 67.0, 65.5, 55.1. HRMS (ESI^+^): Calcd for C_17_H_25_F_2_N_5_O_13_P_2_Br [*M* + H]^+^: 686.0076; Found: 686.0051.

**10n** ^1^H NMR (400 MHz, D_2_O) δ 8.27 (s, 1H), 5.88 (d, *J *=* *5.3 Hz, 1H), 5.16 (5.23, s, 0.4H, 5.09, s, 0.6H), 4.57 (t, *J *=* *5.2 Hz, 1H), 4.44–4.34 (m, 1H), 4.23 (d, *J *=* *3.1 Hz, 1H), 4.17 (s, 3H), 4.12–3.83 (m, 4H,). ^19^F NMR (376 MHz, D_2_O) δ −119.0 (m). ^31^P NMR (162 MHz, D_2_O) δ 4.2 (t, *J *=* *82.2 Hz). ^13^C NMR (101 MHz, D_2_O) δ 155.9, 149.9, 144.4, 139.8, 117.9, 101.2, 96.3, 87.1, 83.8, 82.2, 75.1, 74.4, 70.4, 70.1, 66.7, 65.5. HRMS (ESI^+^): Calcd for C_16_H_23_F_2_N_5_O_13_P_2_Br [*M* + H]^+^: 671.9919; Found: 671.9941.

#### 2.1.3 Synthesis of compounds 13 and 14

Adenosine **1a** (8.01 g, 30 mmol) and KOH (3.6 g, 60 mmol) were added to 100 ml DMF, stirred at room temperature to dissolve the KOH. Then methyl *p*‐toluenesulphonate (6.7 g, 36 mmol) was added dropwise and stirred at room temperature for 12 hr. The solvent was evaporated, and the residue was diluted with CH_3_COCH_3_. The solid was filtered, and the filtrate was separated on silica gel chromatography (CH_2_Cl_2_:MeOH = 20:1) to give a white solid. The white solid was recrystallized with C_2_H_5_OH for three times to give 2′‐OMe‐adenosine as a while solid (1.83 g, yield 22%). The 2′‐OMe‐adenosine (1.83 g, 6.5 mmol) was dissolved in 25 ml dry pyridine, TsCl (1.36 g, 7.1 mmol) was added dropwise at 0°C, then stirred at room temperature for additional 24 hr. The solvent was evaporated, and the residue was separated on silica gel chromatography (CH_2_Cl_2_:MeOH = 50:1) to give **11** as a white solid (0.76 g, yield 27%). The following procedures are the same as route 1. Compound **11** and tris(tetra‐*n*‐butylammonium) hydrogen methylenediphosphonate were reacted in dry CH_3_CN and purified to give 2′‐*O*‐methyl‐5′‐methylenediphosphate adenosine **12** as a light yellow foam solid (yield: 50%). Compounds **12** and **5** were reacted in dry CH_3_CN and purified to give 1″‐*O*‐methyl‐2″,3″‐*O*‐isopropylidene‐2′‐methoxy‐5′‐methylenediphoribose adenosine **13** as a white foam solid (yield: 12%). Compound **13** was treated with aqueous HCl and purified to give 2′‐methoxy‐5′‐methylenediphoribose adenosine **14** as a foam solid (yield: 45%).

**13** ^1^H NMR (400 MHz, D_2_O) δ 8.53 (d, *J *=* *3.1 Hz, 1H), 8.19 (d, *J *=* *6.3 Hz, 1H), 6.11 (dd, *J *=* *5.1, 2.9 Hz, 1H), 4.90 (s, 1H), 4.66 (d, *J *=* *6.0 Hz, 1H), 4.63 (t, *J *=* *4.0 Hz, 1H), 4.50 (d, *J *=* *6.0 Hz, 1H), 4.41 (d, *J *=* *4.0 Hz, 1H), 4.30 (d, *J *=* *2.6 Hz, 1H), 4.18 (t, *J *=* *7.3 Hz, 1H), 4.13 (d, *J *=* *3.9 Hz, 2H), 3.85–3.63 (m, 2H), 3.41 (s, 3H), 3..22 (s, 3H), 2.14 (t, *J *=* *19.9 Hz, 2H), 1.29 (s, 3H), 1.16 (s, 3H). ^31^P NMR (162 MHz, D_2_O) δ 17.8 (m). ^13^C NMR (101 MHz, D_2_O) δ 155.0, 151.9, 148.8, 140.4, 118.6, 112.9, 108.5, 85.5, 85.1, 85.0, 84.5, 84.1, 83.2, 81.1, 68.9, 64.2, 63.6, 58.2, 54.8, 25.1, 23.5. HRMS (ESI^+^): Calcd for C_21_H_34_N_5_O_14_P_2_ [*M* + H]^+^: 626.1628; Found: 626.1622.

**14** ^1^H NMR (400 MHz, D_2_O) δ 8.47 (s, 1H), 8.13 (s, 1H), 6.09 (d, *J *=* *5.8 Hz, 1H), 5.14 (5.25, d, *J *=* *4.1 Hz, 0.4H, 5.12, d, *J *=* *2.0 Hz, 0.6H), 4.67–4.50 (m, 1H), 4.43 (t, *J *=* *5.3 Hz, 1H), 4.34–4.20 (m, 2H), 4.20–4.02 (m, 3H), 4.02–3.83 (m, 3H), 3.41 (s, 3H), 2.13 (td, *J *=* *19.9, 8.4 Hz, 2H). ^31^P NMR (162 MHz, D_2_O) δ 18.0 (m). ^13^C NMR (101 MHz, D_2_O) δ 155.5, 152.8, 148.9, 140.1, 118.5, 101.3, 96.4, 85.3, 84.6, 83.0, 82.0, 75.3, 70.6, 69.0, 64.5, 63.6, 58.2. HRMS (ESI^+^): Calcd for C_17_H_28_N_5_O_13_P_2_ [*M* + H]^+^: 572.1159; Found: 572.1169.

### 2.2 Inhibitory activity and selectivity assay in vitro

#### 2.2.1 Cell culture

The tetracycline‐inducible HEK293 cells stably expressing the human TRPM2,^32^, ^33^ after being induced with 1 μg/ml tetracycline for 12~36 hr, were cultured at 37°C under 5% CO_2_ in DMEM/F‐12 medium (Gibco, USA) containing 10% foetal bovine serum (FBS), 50 units/ml penicillin and 50 mg/ml streptomycin. Human TRPM7 (NCBI Reference Sequence: NM_001301212.1, in pcDNA5/TO vector), TRPM8 (NCBI Reference Sequence: NM_024080.4, in pcDNA4/TO vector), TRPV1 (NCBI Reference Sequence: NM_080704.3, in pcDNA4/TO vector) and TRPV3 (NCBI Reference Sequence: NM_001258205.1, in pcMV6/TO vector) were transfected into HEK‐293 T cells using Lipofectamine 2000 for 24~48 hr before recordings. HEK293 T cells were cultured at 37°C under 5% CO_2_ in DMEM medium (Gibco, USA) containing 10% foetal calf serum (FBS), 100 units/ml penicillin and 100 mg/ml streptomycin. Cells were seeded on coverslips before use.

#### 2.2.2 Western blot

Western blot was conducted as previously described.^34^ In brief, after transfection for 24–36 hr, HEK293 cells were rinsed with ice‐cold PBS and lysed in ice‐cold protein lysis buffer (RIPA buffer, Beyotime Institute of Biotechnology, China) for 30 min. After centrifuging the lysates at 20,817 *g* at 4°C for 10 min, the supernatants were collected and stored at −80°C until use. The protein concentrations were determined using a BCA protein assay kit (Beyotime Institute of Biotechnology, China). Thirty to fifty micrograms of protein/lane was diluted in standard SDS sample buffer and subjected to electrophoresis on 12% SDS‐polyacrylamide gels. Proteins were then transferred to polyvinylidene difluoride membranes (Millipore, USA), blocked with 5% BSA (Sangon Biotech, China) in Tris‐buffered saline containing 0.05% Tween‐20 (TBST) for 2 hr at room temperature and incubated with the primary antibody (TRPM2: Ab11168, Abcam, UK) overnight at 4°C. The membranes were then washed with TBST and incubated with the secondary antibody (goat anti‐rabbit IgG‐HRP: 31420, Thermo Fisher Scientific, USA). Protein bands were visualized using an Odyssey Infrared Imaging System (Li‐Cor Biosciences, USA). Quantity One software (BioRad, USA) was used for densitometric scanning.

#### 2.2.3 Electrophysiology

Patch‐clamp recordings were performed in whole‐cell configuration at room temperature using Axonpatch 200B (Axon, USA) or HEKA EPC10 (HEKA, Germany) amplifier. Similar to our previously reported protocol,^34^ cells were kept in extracellular solution (ECS) containing (in mm): 147 NaCl, 2 KCl, 1 MgCl_2_, 2 CaCl_2_, 10 HEPES and 13 glucose, pH 7.4. Electrodes had a final resistance of 3–5 MΩ when filled with intracellular solution (ICS) containing (in mm): 147 NaCl, 0.05 EGTA, 1 MgCl_2_, 10 HEPES and 0.1 ADPR, pH 7.3. Patch pipettes (2–4 MΩ) were prepared from the Narishige PC‐10 puller (Narishige, Japan) with Borosilicate glass (Sutter, USA).

Test compound was added to either ECS or ICS with a concentration of 0.1 mm, to determine the extracellular or intracellular effect of test compound on TRPM2. The ADPR concentration was fixed at 0.1 mm in the ICS that also contains test compound when tested for intracellular effect. ECS with compound was perfused for at least 60 s before switching to acidic ECS (pH 5.0) that blocks TRPM2 current.

Change of extracellular solution was achieved using an RSC‐160 system (Biologic Science Instruments, France) in which the solution changing time was about 300 ms. Cell was held at 0 mV. Voltage ramps with 500 ms duration from −100 to 100 mV were applied every 5 s. Data were acquired at 10 kHz and filtered offline at 50 Hz. Capacitive currents and series resistance were determined and corrected before each voltage ramp. For analysis, the mean of the first three ramps before channel activation was used for leak‐subtraction of all subsequent current recordings.

For selectivity evaluation, test compound (0.1 mm) was added to the intracellular solution (ICS) to determine intracellular effect of compound on individual TRPM7, TRPM8, TRPV1 and TRPV3, respectively.

For recordings of TRPM7 channels, the extracellular solution (ECS) contains (in mm): 145 NaCl, 2 CaCl_2_, 1 MgCl_2_, 5 KCl, 10 D‐glucose, 10 HEPES. The ICS contains (in mm): 135 CsCl, 10 EGTA, 10 HEPES and 4 CaCl_2_; pH was adjusted to 7.2 with CsOH, osmolarity was adjusted to ~305 mOsm with mannitol. Low concentrations of Ca^2+^ and Mg^2+^ (both in 0.1 mm) were used to activate TRPM7, and high concentrations of Ca^2+^ and Mg^2+^ (2 mm Ca^2+^ and 1 mm Mg^2+^) were used to inhibit TRPM7.

For recordings of TRPM8 and TRPV1, the ECS contains (in mm): 130 NaCl, 5 KCl, 10 D‐glucose, 10 HEPES, 1.2 MgCl_2_ and 1.5 CaCl_2_; pH was adjusted to 7.4 with NaOH. The ICS contains (in mm): 115 CsCl, 10 EGTA, 2 MgCl_2_, 10 HEPES and 5.7 CaCl_2_, pH was adjusted to 7.2 with CsOH, osmolarity was adjusted to ~290 mOsm with mannitol, and the calculated free Ca^2+^ was 200 nm. Menthol (1 mm) was added in the ECS to activate TRPM8, and 2‐APB (0.1 mm) was added in the ECS to inhibit TRPM8. Capsaicin (0.01 mm) and ruthenium red (0.1 mm) were added in the ECS to activate and inhibit TRPV1, respectively.

For TRPV3 recordings, both ECS and ICS contained 130 mm NaCl, 0.2 mm EDTA. 0.1 mm 2‐APB in the ECS was used to activate TRPV3 and was washed out with standard ECS.

### 2.3 Data analysis

All results from patch‐clamp recordings were expressed as mean ± *SEM*, with *n* indicating the number of individual cells from at least three independent experiments. Statistical analysis was performed using two‐tailed paired Student's *t* test for comparison between groups, with *p *<* *.05 considered to be statistically significant. Prism 5 software was used for all statistical analyses.

## 3 RESULTS

### 3.1 Chemistry

**Scheme 1:**
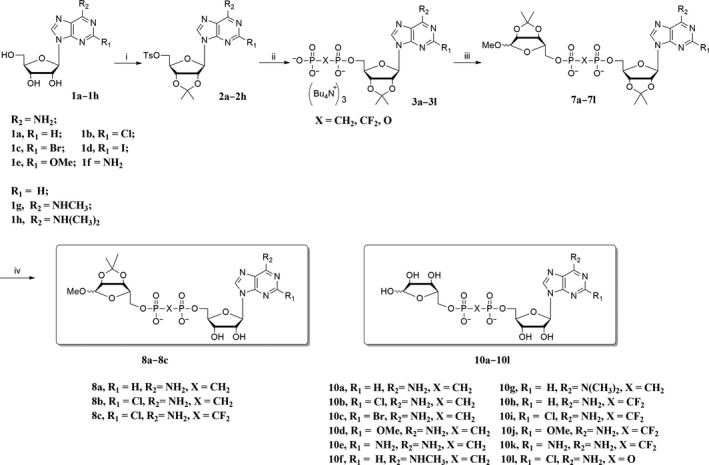
Reagents and conditions: (i) CH_3_ COCH_3_, TsOH·H_2_O, rt, 1 hr, then DCM, TsCl, DMAP, rt, 3~5 hr; (ii) tris(tetra‐*n*‐butylammonium) hydrogen pyrophosphate, dry CH_3_ CN, rt, 48 hr; (iii) 1‐*O*‐methyl‐2,3‐*O*‐isopropylidene‐5‐*O*‐tosyl ribose, rt, dry CH_3_ CN; (iv) 0.8 N HCl, 0°C, 8 hr

**Scheme 2:**
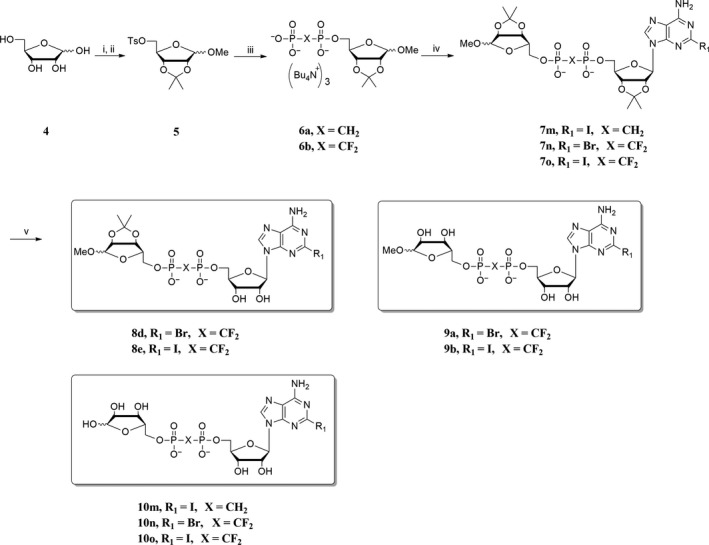
Reagents and conditions: (i) CH_3_ OH, CH_3_ COCl, 0°C, rt, 3 hr; then CH_3_ COCH_3_, TsOH·H_2_O, triethyl orthoformate, rt, overnight; (ii) DCM, TsCl, Et_3_N, rt, 3 hr; (iii) tris(tetra‐*n*‐butylammonium) hydrogen pyrophosphate, dry CH_3_ CN, rt, 24 hr; (iv) **2c** or **2d**, dry CH_3_ CN, rt, 36 hr; (v) 0.8 N HCl, 0°C, 8 hr

**Scheme 3:**
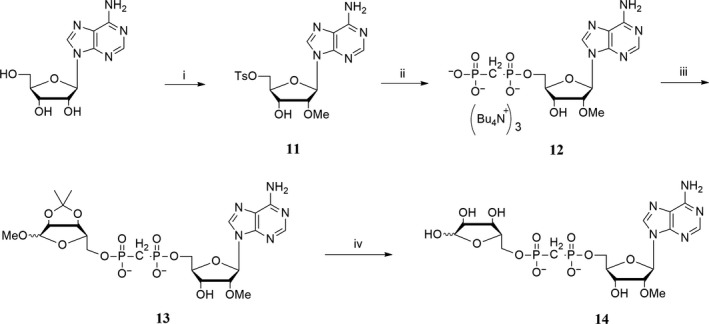
Reagents and conditions: (i) methyl *p*‐toluenesulphonate, KOH, DMF, rt, 12 hr; then Py, TsCl, rt, 24 hr; (ii) tris (tetra‐*n*‐butylammonium) hydrogen methylene phosphate, dry CH_3_ CN, rt, 16 hr; (iii) 2,3‐*O*‐isopropylidene‐5‐*O*‐tosyl ribose **5**, dry CH_3_ CN, rt, 48 hr; (iv) 0.8 N HCl, 0°C, 8 hr

All the ADPR analogues (Table 1; Figure 2) were synthesized in two simple routes (Figure 3; Schemes 1, 2, 3). ADP analogues and RDP analogues were key intermediates for the synthesis of protected ADPR analogues. The protected ADPR analogues were deprotected with aqueous HCl to give final ADPR analogues.

**Table 1:**
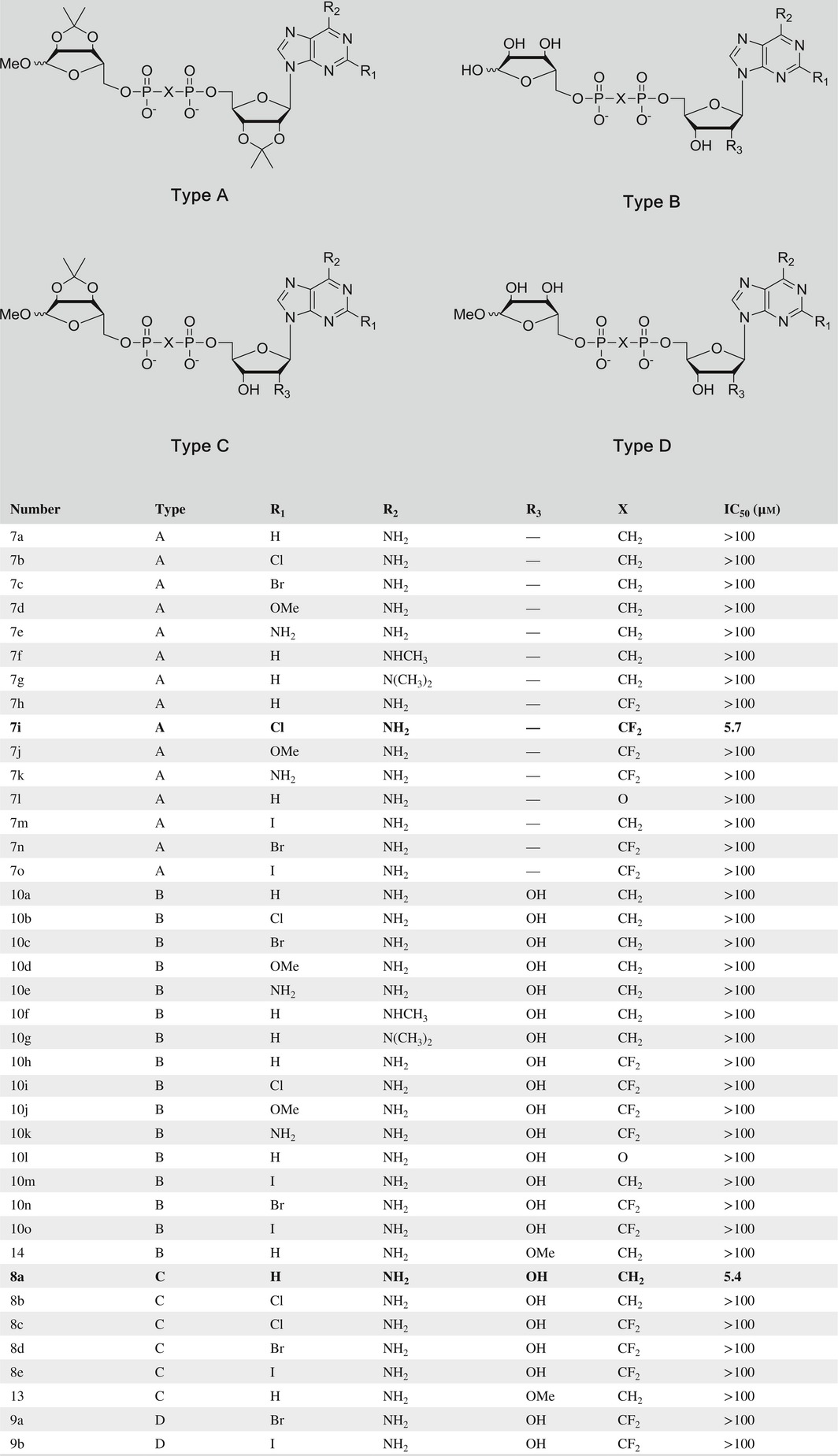
Structure and activity of all synthesized ADPR analogues

In route 1, C‐2 and C‐6 position substituted adenosines were treated with acetone in acidic solution, then reacted with tosyl chloride to give 2′,3′‐*O*‐isopropylidene‐5′‐*O*‐tosyl adenosines **2a~2h** in moderate yield (45%~80%) (Scheme 1). Compounds **2a~2h** were phosphorylated with corresponding tris(tetra‐*n*‐butylammonium) hydrogen pyrophosphate to give ADP analogues **3a~3l** in moderate yield (50%~70%). Compounds **3a~3l** were esterified with 1‐*O*‐methyl‐2,3‐*O*‐isopropylidene‐5‐*O*‐tosyl ribose **5** to give protected ADPR analogues **7a~7l**. Finally, the protected ADPR analogues were deprotected with 0.8 N aqueous HCl and purified by HPLC to give ADPR analogues **8a~8c**,** 10a~10l**.

Applying route 1 gave compound **7c** in low yield (yield <10%). Thus, route 2 was designed (Scheme 2), in which 1‐*O*‐methyl‐2,3‐*O*‐isopropylidene‐5‐*O*‐tosyl ribose **5** instead of 2′,3′‐*O*‐isopropylidene‐5′‐*O*‐tosyl adenosine was phosphorylated with corresponding tris(tetra‐*n*‐butylammonium) hydrogen pyrophosphate to give RDP analogue **6a**,** 6b** in moderate yield (70%). Compounds **6a**,** 6b** were esterified with **2c** or **2d** to give protected ADPR analogues **7m~7o**. Compounds **7m~7o** were deprotected to give **8d**,** 8e**,** 9a**,** 9b**,** 10m~10o**. ADPR analogues **13** and **14** were synthesized with similar procedures to route 1 (Scheme 3), in which 2′‐*O*‐methyl‐5′‐*O*‐tosyl adenosine **11** was synthesized from reagent **1a**, treated with methyl *p*‐toluenesulphonate and tosyl chloride. In Moreau's report,^28^ ADPR analogues are synthesized using NADase as an important catalyst, and quite a number of individual synthetic routes were applied. Taken together, in this study, a more efficient way for synthesis of ADPR analogues was developed.

### 3.2 Intracellular, but not extracellular, inhibition of TRPM2 by two novel ADPR analogues 7i and 8a

**Figure 4:**
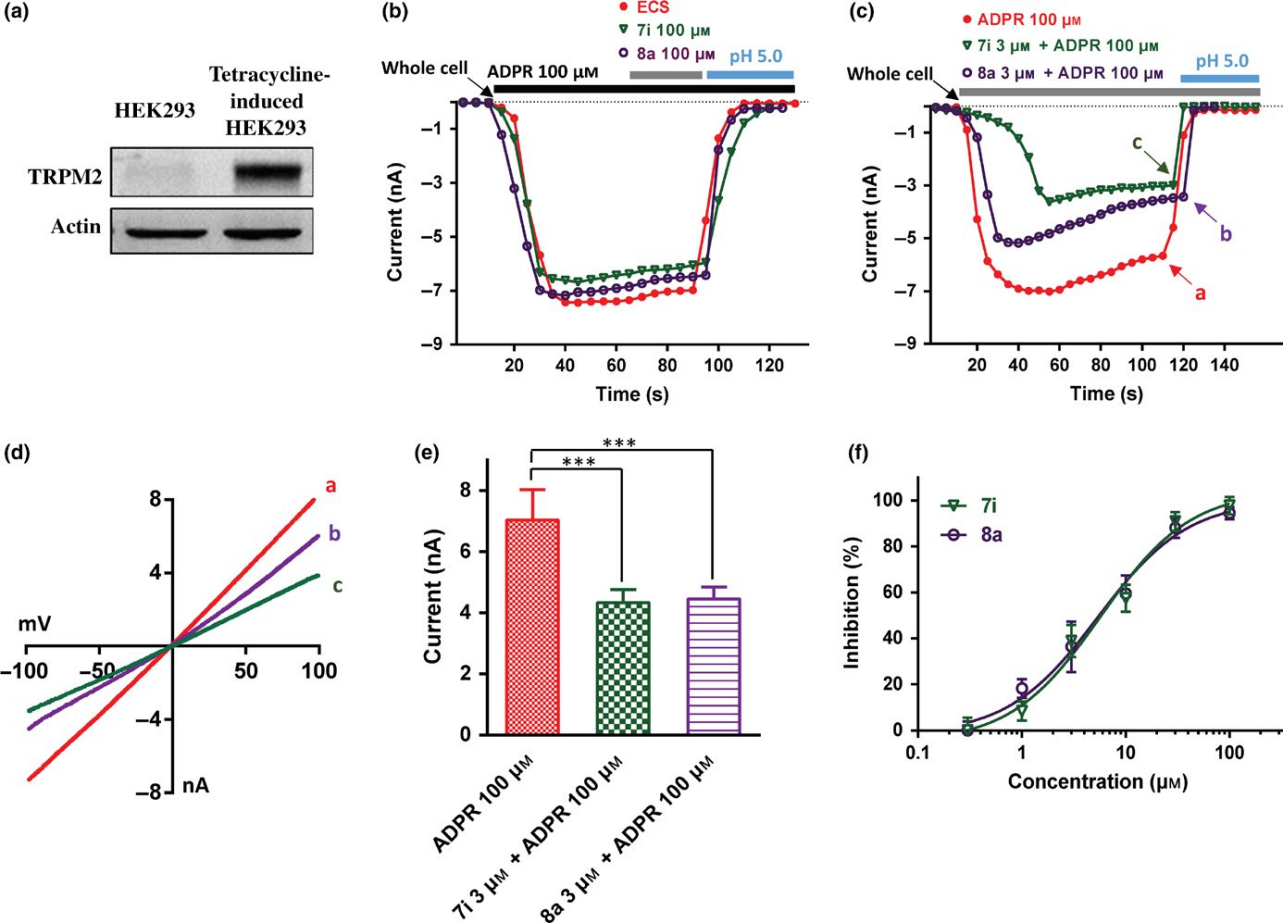
The concentration‐dependent inhibition of TRPM2 by compounds **7i** and **8a**. (a) TRPM2 channel protein expression in HEK293 and tetracycline‐induced HEK293 cells by Western blot. (b) Representative currents of 100 μm ADPR‐induced TRPM2 with the extracellular treatments of ECS (red), 100 μm **7i** (green) and 100 μm **8a** (purple) showed no inhibitory activity. (c) Representative currents of 100 μm ADPR alone (red), ADPR mixed with 3 μm **7i** (green) and ADPR mixed with 3 μm **8a** (purple) showed inhibitory activity on TRPM2 activation. The IC_50_ for the two compounds is approximately about 3 μm. (d) I–V relationship curves showing the time‐point a, b and c from panel (c) before switch to pH 5.0. (e) Mean peak currents from recordings of (b), *n* = 5 for ADPR group, *n* = 6 for ADPR + **7i** group, and *n* = 5 for ADPR + **8a** group; ***p <* *.005. (f) Concentration–response relationship of compounds **7i** (green) and **8a** (purple), *n* = 3. Shaded bar in grey and cyan (for b & c) indicates the perfusion of either **7i** or **8a**, and the washout, respectively [Colour figure can be viewed at wileyonlinelibrary.com]

To screen for TRPM2 inhibitors, the effects of all synthesized ADPR analogues (Table 1) were evaluated on TRPM2 channels stably expressed in HEK293 cells by whole‐cell patch‐clamp recordings. In HEK293 cells, the expression of TRPM2 channel proteins was detected by Western blot analysis (Figure 4a). It is known that TRPM2 is only activated by intracellular ADPR that binds directly to TRPM2 channel's enzymatic NUDT9‐H domain in the C‐terminal tail.^6^ We tested whether extracellular administration of individual ADPR analogues had any effect on TRPM2 currents (Table S1). When administered extracellularly (100 μm), none of the synthesized ADPR analogues showed any inhibition on TRPM2 currents induced by intracellular ADPR, in which compounds **7i** and **8a** serving as representatives (Figure 4b). In contrast, intracellular administration of **7i** or **8a** at 3 μm resulted in a reduction of TRPM2 current by 39% and 36%, respectively, as compared with 100 μm ADPR as a control (Figure 4c–e, *p* < .005). Applying different concentrations of **7i** or **8a** gave rise to a dose‐dependent inhibition of TRPM2 current with IC_50_s for **7i** at 5.7 μm and **8a** at 5.4 μm (Figure 4f). These results suggest compounds **7i** and **8a** inhibited TRPM2 intracellularly, possibly through competing with ADPR by binding to the intracellular NUDT9‐H domain.

### 3.3 Lack of inhibitory effect on other TRP channels by compound 7i or 8a

**Figure 5:**
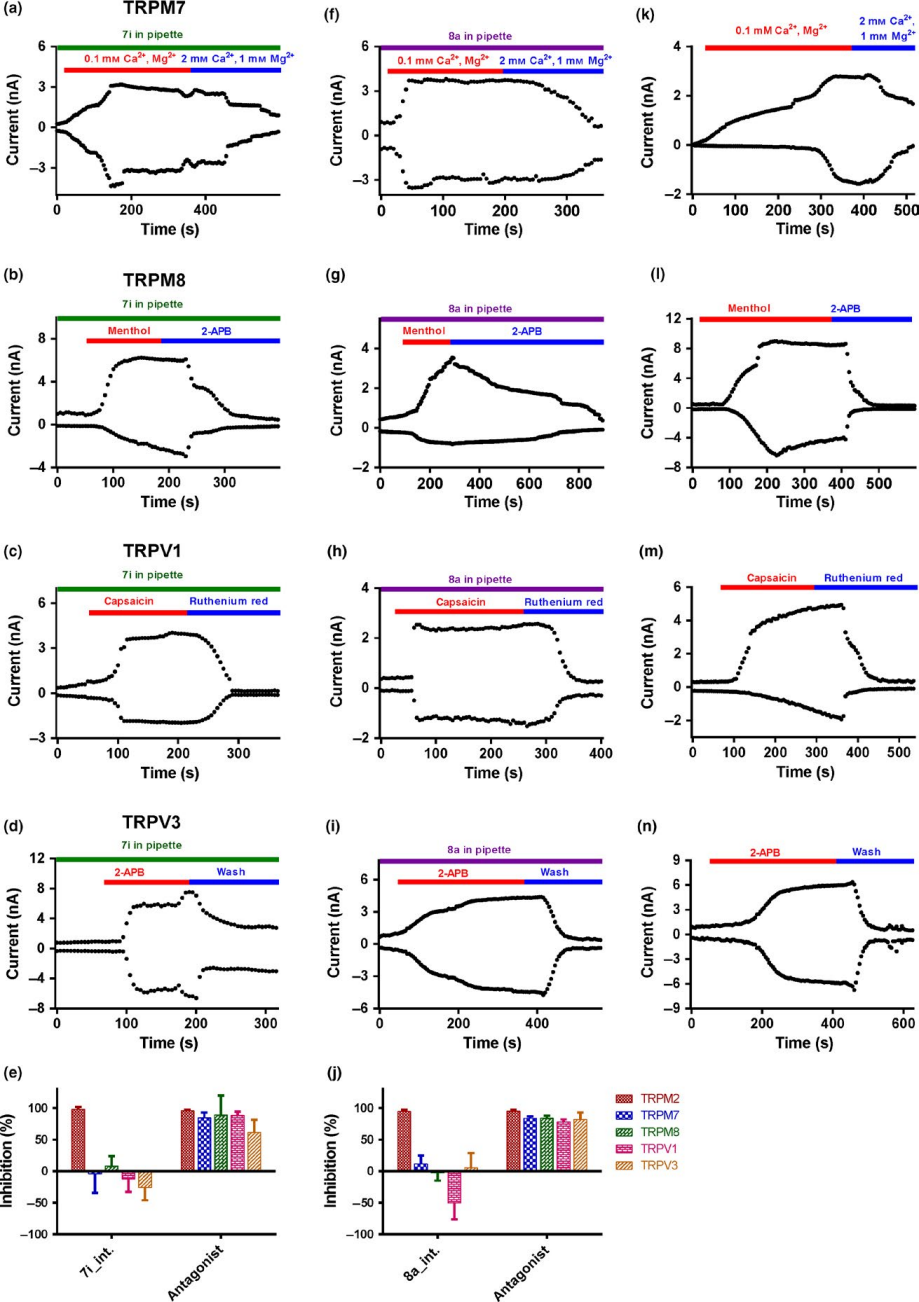
Selectivity evaluations of intracellular 100 μm **7i** (a–d, *n* = 3) or 100 μm **8a** (f–i, *n* = 3) in the pipette on other TRP channels (TRPM7, TRPM8, TRPV1, TRPV3). (k–n) The effect of controls with their corresponding agonist on the other TRP channels (TRPM7, TRPM8, TRPV1, TRPV3, *n* = 4), without **7i** or **8a** in pipette. (e) Summary of the effect of compound **7i** on TRP channels (*n* = 3), compared with their corresponding inhibitor (100 μm 2‐APB for TRPM2 and TRPM8; 2 mm of Ca^2+^ and 1 mm Mg^2+^ for TRPM7; 100 μm ruthenium red for TRPV1; and washout with bath solution for TRPV3). (j) Summary for the effect of compound **8a** on TRP channels (*n* = 3), compared with their corresponding inhibitor (100 μm 2‐APB for TRPM2 and TRPM8; 2 mm of Ca^2+^ and 1 mm Mg^2+^ for TRPM7; 100 μm ruthenium red for TRPV1; and washout with bath solution for TRPV3) [Colour figure can be viewed at wileyonlinelibrary.com]

To evaluate the compounds selectivity, the effects of both **7i** and **8a** on other TRP channels, including the closely related channels TRPM7 and TRPM8, and the more distantly related channels TRPV1 and TRPV3, were tested. TRPM7 currents overexpressed in HEK293T cells were activated by low concentrations of Ca^2+^ and Mg^2+^ (both in 0.1 mm) in the extracellular solution (ECS). Compared with the control without **7i** (Figure 5k), the intracellular administration of **7i** (100 μm) showed no effect on TRPM7 currents (Figure 5a). Similarly, adding 100 μm **7i** compound in the pipette did not affect TRPM8 pharmacology in response to either activator, menthol (1 mm) or blocker 2‐APB (100 μm) (Figure 5b,l). Adding compound **7i** (100 μm) in the pipette had no effect on activating TRPV1 induced by capsaicin, although appeared to delay the channel activation (Figure 5c,m). The intracellular effect of compound **7i** on TRPV3 was also tested. As shown in Figure 5d,n, application of compound **7i** (100 μm) in pipette had no effect on TRPV3 currents activated by 100 μm 2‐APB. These results show that intracellular compound **7i** has no inhibitory effect on TRPM7, TRPM8, TRPV1 and TRPV3 (Figure 5a–e).

Similarly, the effects of compound **8a** on TRPM7, TRPM8, TRPV1 and TRPV3 were also tested. Intracellular administrations of compound **8a** (100 μm) did not show any inhibitory effect on these TRP channels (Figure 5f–j,k–n). Taken together, these results indicate that compounds **7i** and **8a** are specific in inhibiting TRPM2 channel without affecting other TRP channels.

### 3.4 Structure–activity relationship (SAR) of synthesized ADPR analogues for TRPM2 inhibition

**Figure 6:**
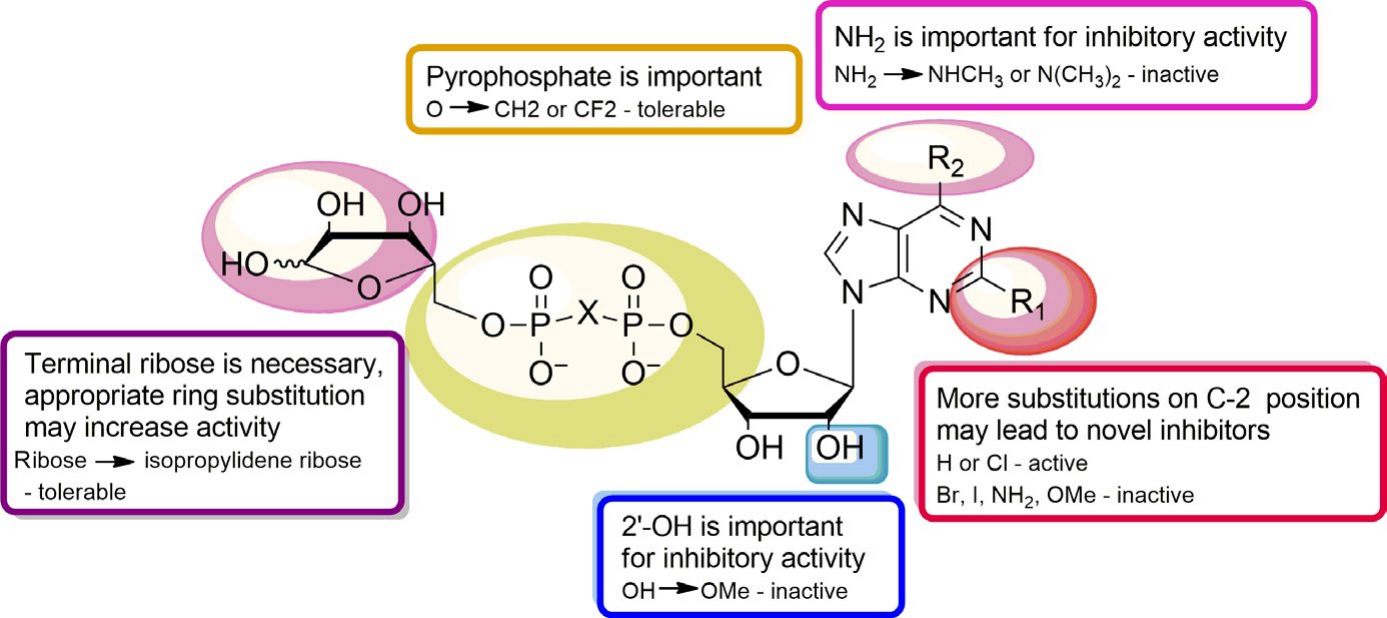
Structure–activity relationship of ADPR‐analogue inhibitor. Purine C‐2 position (R1, red plaque): appropriate substitutions may contribute to inhibitory activity; purine C‐6 position (R2, pink plaque): amino is important for inhibitory activity; ribose 2′‐position (blue plaque): hydroxyl is important for inhibitory activity; pyrophosphate (yellow plaque): pyrophosphate is important, but appropriate modifications are tolerable; terminal ribose (purple plaque): ribose is necessary, and appropriate ring substitution may increase activity [Colour figure can be viewed at wileyonlinelibrary.com]

Totally, 39 compounds were synthesized to explore the structure–activity relationship (SAR) of the ADPR analogues (Figure 6). To begin with adenosine modification, ADPR analogues (**7h**,** 7g**,** 10h**,** 10g**) with 6‐methylamino or 6‐dimethylamino substitution showed no inhibitory activity, indicating the necessity of 6‐amino for inhibitory activity. This is in accordance with Moreau's report, in which 6‐oxygen substituted ADPR (IDPR) shows no inhibitory activity.^28^ Substitutions at C‐2 position of purine base showed different influences. Compounds **7i** (2‐chloro) and **8a** (2‐hydrogen) exhibited moderate activity, while other substitutions such as 2‐bromo, 2‐iodo, 2‐amino and 2‐methoxy (**7a~7e**,** 7h**,** 7j~7o**,** 10a~10o**) did not. Thus, more kinds of substitutions should be investigated to further reveal the effect of C‐2 position modification on inhibitory activity, which may lead to novel potent inhibitors. As to adenosine ribose part, neither compound **13** nor compound **14** with 2′‐methoxy substitution showed any inhibitory activity indicating the importance of C‐2′ position for analogues binding.

With regard to pyrophosphate, both two active compounds **7i** and **8a** possess a substituted pyrophosphate (O → CH_2_ or CF_2_). In contrast, inactive compound **7o** reserves oxygen‐linked pyrophosphate. Previous report considered pyrophosphate as an activity‐keeping group and none of the sulphonamide, squarate or triazole substituted ADPR analogues showed any activity.^28^ Therefore, appropriate modifications on the pyrophosphate can be tolerable.

As to the terminal ribose, it has been shown that a five‐membered ring but the hydroxyl groups of the terminal ribose plays a critical role in filling the binding site.^28^ Here, compounds **7i** and **8a** (both terminal ribose hydroxyl protected) showed inhibitory activity, while their corresponding deprotected products **10i** and **10a** lost inhibitory activity. Taken together, we suppose, applying saturated ring mimicking essential terminal ribose is feasible, which may improve the inhibitory activity.

## 4 DISCUSSION

Searching for selective TRPM2 inhibitors as tool molecules is critical for probing pharmacological functions of the channel. Up to date, there are non‐specific TRPM2 inhibitors such as 2‐APB, clotrimazole and FFA. These inhibitors exhibit low potency on TRPM2 channel and also are lack of selectivity on other TRP channels. Moreau et al. previously reported several ADPR analogues as specific TRPM2 inhibitors without interfering Ca^2+^ release induced by cADPR, NAADP or IP_3_.^28^ However, the selectivity of these analogues against other TRP channels remains unclear.

To discover novel TRPM2 selective inhibitors, we systematically synthesized a new series of ADPR analogues (Schemes 1, 2, 3) in this study. Modifications were mainly focused on pyrophosphate linkage and adenosine, to improve the potency and membrane permeability. Two major routes were employed for ADPR analogue synthesis (Figure 3). Both synthetic routes are relative simple, of which ADP analogues and RDP analogues were the key intermediates (Schemes 1, 2, 3). These two key intermediates were obtained in moderate yield (50%~70%) and can be employed for ADPR analogues derivatization easily. Compared with previous report of which ADPR analogues are synthesized through quite a number of individual synthetic routes,^28^ a more efficient way for synthesis of ADPR analogues was developed in this study, though esterification and deprotection steps give the desired products (protected ADPR analogues and ADPR analogues) in low yield.

Totally 39 new ADPR analogues were synthesized. Compounds **7i** and **8a** were confirmed as TRPM2 inhibitors with moderate activity, exerting intracellular inhibition of TRPM2 current, and demonstrating selectivity over other TRP members including TRPM7, TRPM8, TRPV1 and TRPV3. The characters of these two inhibitors probably attribute to the nature of ADPR analogues. It is well known that ADPR gates TRPM2 through binding to the cytosolic C‐terminal NUDT9‐H domain which is a unique structure domain among TRP channels.^5^ The two inhibitors **7i** and **8a** likely compete with ADPR for binding to the NUDT9‐H domain, serving as intracellular inhibitors. These two new inhibitors are firstly identified TRP‐subtype selective TRPM2 inhibitors, which are selective over TRPM7, TRPM8, TRPV1 and TRPV3, providing new tool molecules for probing pharmacological functions of TRPM2 channel. Previous reported nonselective inhibitors such as FFA and 2‐APB also show significant effects on other ion channels. FFA can also inhibit TRPM3, TRPM4 and TRPM5,^35^, ^36^ while 2‐APB also activates TRPV1, TRPV2 and TRPV3.^25^ When employing selective inhibitors **7i** or **8a** on probing TRPM2 function, these unwanted side‐effects will be eliminated, so that the results will be more relevant to TRPM2 channel. Though the two inhibitors still exhibit poor membrane permeability limiting their extracellular application in probing TRPM2 function, applying liposomes or nucleotide prodrug technique will allow the transport of **7i** or **8a** across the membrane. These newly identified TRP‐subtype selective inhibitors can serve as new pharmacological tools for probing TRPM2 channel.

What is more, the identification of ADPR‐analogue‐based TRPM2 inhibitors provides further insights into optimizing potency and membrane permeability. With regarding to improving membrane permeability, none of the ADPR analogues show any inhibitory activity when administered extracellularly; thus, it is still insufficient though substituting pyrophosphate oxygen with methylene or difluoromethylene. Recently, a neutral triazole substituted cADPR (P_2_O_6_^−^ → triazole) that retains its ability to activate Ca^2+^ release was reported by Swarbrick et al.^37^ This encourages employing more kinds of neutral substitutes, though the pyrophosphate group seems critical for keeping the activity. For potency optimizing, more kinds of substitutions at C‐2 position should be investigated, which may lead to novel potent inhibitors. Other successful cases that took purine C‐2 position substitution support this hypothesis. Fischer et al. reported 2‐chloro‐adenosine‐5′‐*O*‐α‐boranodiphosphate (2‐Cl‐α‐BH_3_‐ADP) as a potent agonist (EC_50_ = 7 nm) of P2Y1 receptor, whose endogenous substrate is adenosine diphosphate (ADP).^38^ Similarly, 2‐methylthio‐AMP was reported as an inhibitor of P2Y12 receptor, for which ADP serves as the endogenous substrate as well.^39^, ^40^ As far as to terminal ribose, neither ribose nor isopropylidene‐protected ribose determines the inhibitory activity, thus employing more kinds of rings is an alternative way.

## 5 CONCLUSIONS

Searching for selective TRPM2 inhibitors as tool molecules is critical for probing pharmacological functions of the channel. In this study, we synthesized 39 new ADPR analogues, of which the modifications were mainly focused on pyrophosphate linkage and adenosine. Their effects on TRPM2 channel were evaluated. Compounds **7i** and **8a** were confirmed as TRPM2 inhibitors with moderate activity, exerting intracellular inhibition of TRPM2 current, and demonstrating selectivity over other TRP members including TRPM7, TRPM8, TRPV1 and TRPV3. Besides, a more sufficient SAR of ADPR‐analogue TRPM2 inhibitors was summarized in this study.

In summary, we synthesized and obtained two novel subtype selective TRPM2 inhibitors. The newly identified TRPM2 inhibitors can serve as new pharmacological tools for further investigation and validation of TRPM2 channel as a drug target. The summarized SAR may also provide insights into further improving existing inhibitors as potential lead compounds.

## CONFLICT OF INTEREST

All authors declare that there is no conflict of interest in this study.

## AUTHOR CONTRIBUTIONS

X. Luo carried out the chemical synthetic experiments. K. Y. Zhan and W. Yang conducted the activity assay experiments. M. Li conducted the inhibitory selectivity experiments. X. Luo, M. Li and K. Y. Zhan analysed the data. X. Luo drafted the manuscript. K. W. Wang, P. L. Yu, W. Yang, L. H Zhang and L. R. Zhang conceived and supervised the project. K. W. Wang, P. L. Yu, W. Yang and L. R. Zhang participated in data analysis and wrote and finalized the manuscript writing.
