# Supplementary material for: Selective inhibition of TRPM2 channel by two novel synthesized ADPR analogues
Source: Chem Biol Drug Des. 2017 Nov 15;91(2):552–66. doi: 10.1111/cbdd.13119 (PMC5813235; doi:10.1111/cbdd.13119)
Supplement: Supplementary file 1 [file CBDD-91-552-s001.doc]

**Supporting information**

**Selective inhibition of TRPM2 channel by two novel synthesized ADPR analogues**

Xiao Luo^1^, Meng Li^2^, Kaiyu Zhan^3^, Wei Yang^3^, Lihe Zhang^1^, KeWei Wang^2,4*^, Peilin Yu^5*^, and Liangren Zhang^1*^

^1^State Key Laboratory of Natural and Biomimetic Drugs, Peking University, Beijing 100191, China; ^2^Department of Neurobiology, Neuroscience Research Institute, Peking University Health Science Center, Peking University School of Pharmaceutical Sciences , Beijing 100191, China; ^3^Department of Neurobiology, Zhejiang University School of Medicine, Hangzhou, Zhejiang, 310058, China; ^4^Department of Pharmacology, School of Pharmacy, Qingdao University, Qingdao 266021, China; ^5^Department of Toxicology, School of Public Health, Zhejiang University, Hangzhou, Zhejiang, 310058, China

**1. The effect of all the ADPR analogues on TRPM2 currents induced by ADPR**

Table S**1**. The effect of all the ADPR analogues on TRPM2 currents induced by ADPR

| **Number** | **Extracellularly assay (nA)** | ***SD*** | **Inracellularly assay (nA)** | ***SD*** |
| --- | --- | --- | --- | --- |
|
| **ADPR** | **-5.74** | ***0.30*** | **-5.84** | ***0.27*** |
| 7a | -4.60 | *0.43* | -5.63 | *0.83* |
| 7b | -5.75 | *0.39* | -6.25 | *0.61* |
| 7c | -5.73 | *0.60* | -4.79 | *0.17* |
| 7d | -5.16 | *0.36* | -5.59 | *0.43* |
| 7e | -5.25 | *0.55* | -4.90 | *0.23* |
| 7f | -5.63 | *0.14* | -4.49 | *0.47* |
| 7g | -5.28 | *0.22* | -5.94 | *0.31* |
| 7h | -5.66 | *0.25* | -5.43 | *0.41* |
| **7i** | **-5.83** | ***0.19*** | **-0.02** | ***0.02*** |
| 7j | -5.34 | *0.41* | -4.88 | *0.17* |
| 7k | -5.80 | *0.39* | -5.08 | *0.22* |
| 7l | -6.09 | *0.15* | -5.26 | *0.12* |
| 7m | -6.05 | *0.59* | -5.94 | *0.70* |
| 7n | -5.39 | *1.06* | -4.65 | *0.27* |
| 7o | -4.43 | *0.32* | -4.65 | *0.06* |
| 13 | -5.41 | *0.36* | -5.14 | *0.53* |
| **8a** | **-4.83** | ***0.18*** | **-0.26** | ***0.12*** |
| 8b | -5.22 | *0.57* | -4.55 | *0.30* |
| 8c | -5.32 | *0.52* | -5.03 | *0.05* |
| 8d | -6.07 | *0.825* | -5.86 | *0.21* |
| 8e | -5.40 | *0.46* | -4.87 | *0.22* |
| 9a | -4.74 | *0.40* | -5.85 | *0.25* |
| 9b | -6.21 | *0.35* | -4.49 | *0.15* |
| 10a | -5.17 | *0.30* | -5.30 | *0.16* |
| 10b | -5.79 | *0.36* | -5.82 | *0.24* |
| 10c | -5.90 | *0.25* | -5.56 | *0.23* |
| 10d | -5.90 | *0.61* | -5.37 | *0.35* |
| 10e | -6.15 | *0.41* | -5.61 | *0.01* |
| 10f | -4.84 | *0.33* | -5.87 | *0.45* |
| 10g | -5.38 | *0.32* | -5.93 | *0.19* |
| 10h | -5.93 | *0.62* | -4.17 | *0.39* |
| 10i | -4.56 | *0.15* | -4.64 | *0.26* |
| 10j | -6.11 | *0.20* | -4.90 | *0.32* |
| 10k | -5.82 | *0.53* | -5.88 | *0.62* |
| 10l | -5.31 | *0.14* | -5.83 | *0.69* |
| 10m | -6.17 | *0.52* | -5.80 | *0.12* |
| 10n | -5.59 | *0.26* | -5.74 | *0.33* |
| 10o | -5.22 | *0.32* | -4.50 | *0.42* |
| 14 | -5.43 | *0.59* | -5.20 | *0.46* |

**2. Characterization data by ^1^H, ^13^C NMR, MS and HRMS for all compounds**

**2’,3’-*O*-Isopropylidene-5’-*O*-tosyladenosine (2a)**

White solid; yield: 76%.

^1^H NMR (400 MHz, CDCl_3_) δ 8.23 (s, 1H), 7.85 (s, 1H), 7.62 (d, *J* = 8.5 Hz, 2H), 7.18 (d, *J* = 8.5 Hz, 2H), 6.06 (d, *J* = 2.3 Hz, 1H), 6.03 (s, 2H), 5.34 (dd, *J* = 6.2, 2.3 Hz, 1H), 5.06 (dd, *J* = 6.2, 3.1 Hz, 1H), 4.49 (dd, *J* = 8.9, 4.3 Hz, 1H), 4.27 (ddd, *J* = 15.9, 10.0, 5.1 Hz, 2H), 2.39 (s, 3H), 1.59 (s, 3H), 1.37 (s, 3H). ^13^C NMR (101 MHz, CDCl_3_) δ 155.6, 153.0, 149.0, 145.0, 139.7, 132.2, 129.6, 127.8, 120.2, 114.6, 91.0, 84.9, 84.2, 81.6, 69.2, 27.1, 25.3, 21.7. ESI-MS m/z: 462 [M+H]^+^.

**2’,3’-*O*-Isopropylidene-5’-*O*-tosyl-2-chloroadenosine (2b)**

White solid; yield: 80%.

^1^H NMR (400 MHz, CDCl_3_) δ 7.86 (s, 1H), 7.66 (d, *J* = 8.3 Hz, 2H), 7.19 (d, *J* = 8.2 Hz, 2H), 6.62 (s, 2H), 6.05 (d, *J* = 2.1 Hz, 1H), 5.22 (dd, *J* = 6.2, 2.1 Hz, 1H), 5.00 (dd, *J* = 6.2, 3.2 Hz, 1H), 4.48 (dd, *J* = 8.4, 5.0 Hz, 1H), 4.30 (t, *J* = 5.1 Hz, 2H), 2.38 (s, 3H), 1.59 (s, 3H), 1.36 (s, 3H). ^13^C NMR (101 MHz, CDCl_3_) δ 156.4, 154.1, 150.0, 145.1, 139.9, 132.1, 129.7, 127.9, 118.9, 114.8, 90.6, 84.9, 84.3, 81.4, 69.3, 27.1, 25.3, 21.7. ESI-MS m/z: 496 [M+H]^+^.

**2’,3’-*O*-Isopropylidene-5’-*O*-tosyl-2-bromoadenosine (2c)**

White solid; yield: 77%.

^1^H NMR (400 MHz, CDCl_3_) δ 7.83 (s, 1H), 7.67 (d, *J* = 8.3 Hz, 2H), 7.18 (d, *J* = 8.1 Hz, 2H), 6.54 (s, 2H), 6.04 (d, *J* = 2.0 Hz, 1H), 5.20 (dd, *J* = 6.2, 2.0 Hz, 1H), 5.00 (dd, *J* = 6.2, 3.3 Hz, 1H), 4.48 (dd, *J* = 8.5, 5.1 Hz, 1H), 4.30 (d, *J* = 5.2 Hz, 2H), 2.38 (s, 3H), 1.59 (s, 3H), 1.36 (s, 3H). ^13^C NMR (101 MHz, CDCl_3_) δ 156.1, 149.8, 145.1, 144.8, 139.8, 132.2, 129.6, 127.9, 119.3, 114.8, 90.6, 85.0, 84.4, 81.4, 69.3, 27.1, 25.4, 21.7. ESI-MS m/z: 540 [M+H]^+^.

**2’,3’-*O*-Isopropylidene-5’-*O*-tosyl-2-iodoadenosine (2d)**

White solid; yield: 72%.

^1^H NMR (400 MHz, CDCl_3_) δ 7.75 (s, 1H), 7.67 (d, *J* = 8.3 Hz, 2H), 7.17 (d, *J* = 8.0 Hz, 2H), 6.38 (s, 2H), 6.02 (d, *J* = 1.9 Hz, 1H), 5.17 (dd, *J* = 6.2, 1.9 Hz, 1H), 4.99 (dd, *J* = 6.2, 3.4 Hz, 1H), 4.48 (dt, *J* = 6.2, 4.3 Hz, 1H), 4.36 – 4.23 (m, 2H), 2.37 (s, 3H), 1.58 (s, 3H), 1.36 (s, 3H). ^13^C NMR (101 MHz, CDCl_3_) δ 155.3, 149.1, 148.8, 145.0, 139.5, 132.2, 129.6, 128.0, 119.6, 114.7, 90.6, 85.3, 84.5, 81.4, 69.3, 27.1, 25.4, 21.7. ESI-MS m/z: 610 [M+Na]^+^.

**2’,3’-*O*-Isopropylidene-5’-*O*-tosyl-2-methoxyadenosine (2e)**

White solid; yield: 75%.

^1^H NMR (400 MHz, CDCl_3_) δ 7.64 (s, 1H), 7.62 (d, *J* = 8.2 Hz, 2H), 7.17 (d, *J* = 8.2 Hz, 2H), 5.97 (d, *J* = 2.0 Hz, 1H), 5.55 (s, 2H), 5.37 (dd, *J* = 6.3, 2.0 Hz, 1H), 5.04 (dd, *J* = 6.3, 3.3 Hz, 1H), 4.43 (dd, *J* = 8.7, 5.3 Hz, 1H), 4.25 (dd, *J* = 5.4, 1.7 Hz, 2H), 3.90 (s, 3H), 2.39 (s, 3H), 1.58 (s, 3H), 1.36 (s, 3H). ^13^C NMR (101 MHz, CDCl_3_) δ 162.3, 156.5, 150.4, 145.1, 138.5, 132.2, 129.6, 127.8, 116.5, 114.6, 90.5, 84.5, 83.9, 81.4, 69.1, 54.7, 27.1, 25.3, 21.7. ESI-MS m/z: 492 [M+H]^+^.

**2’,3’-*O*-Isopropylidene-5’-*O*-tosyl-2-aminoadenosine (2f)**

White solid; yield: 45%.

^1^H NMR (400 MHz, CDCl_3_) δ 7.71 (d, *J* = 8.1 Hz, 1H), 7.53 (s, 2H), 7.23 (d, *J* = 8.1 Hz, 2H), 5.91 (s, 1H), 5.68 (s, 2H), 5.35 (d, *J* = 6.2 Hz, 1H), 5.25 – 5.10 (m, 1H), 4.99 (s, 2H), 4.64 (dd, *J* = 9.9, 6.5 Hz, 1H), 4.43 (d, *J* = 2.1 Hz, 1H), 4.09 (m, 1H), 2.40 (s, 3H), 1.58 (s, 3H), 1.38 (s, 3H). ^13^C NMR (101 MHz, CDCl_3_) δ 159.8, 156.0, 150.9, 145.1, 137.4, 132.3, 129.7, 127.9, 115.0, 114.3, 91.0, 84.6, 84.0, 82.0, 68.7, 27.0, 25.3, 21.6. ESI-MS m/z: 477 [M+H]^+^.

**2’,3’-*O*-Isopropylidene-5’-*O*-tosyl-6-methylaminoadenosine (2g)**

White solid; yield: 76%.

^1^H NMR (400 MHz, CDCl_3_) δ 8.28 (s, 1H), 7.78 (s, 1H), 7.64 (d, *J* = 8.2 Hz, 2H), 7.18 (d, *J* = 8.2 Hz, 2H), 6.05 (d, *J* = 1.9 Hz, 1H), 5.91 (s, 1H), 5.35 (dd, *J* = 6.2, 1.9 Hz, 1H), 5.06 (dd, *J* = 6.2, 3.0 Hz, 1H), 4.48 (dd, *J* = 6.2, 3.3 Hz, 1H), 4.28 (ddd, *J* = 17.0, 10.6, 5.5 Hz, 2H), 3.23 (m, 3H), 2.40 (s, 3H), 1.60 (s, 3H), 1.38 (s, 3H). ^13^C NMR (101 MHz, CDCl_3_) δ 155.5, 153.1, 150.6, 145.0, 139.0, 132.3, 129.6, 128.6, 127.8, 114.5, 91.0, 84.8, 84.2, 81.7, 69.2, 60.4, 27.0, 25.3, 21.6. ESI-MS m/z: 476 [M+H]^+^.

**2’,3’-*O*-Isopropylidene-5’-*O*-tosyl-6-dimethylaminoadenosine (2h)**

White solid; yield: 77%.

^1^H NMR (400 MHz, CDCl_3_) δ 8.19 (s, 1H), 7.73 (s, 1H), 7.64 (d, *J* = 8.0 Hz, 2H), 7.17 (d, *J* = 8.0 Hz, 2H), 6.03 (s, 1H), 5.32 (d, *J* = 5.5 Hz, 1H), 5.06 (d, *J* = 3.0 Hz, 1H), 4.46 (s, 1H), 4.34 – 4.21 (m, 2H), 3.54 (s, 6H), 2.39 (s, 3H), 1.59 (s, 3H), 1.37 (s, 3H). ^13^C NMR (101 MHz, CDCl_3_) δ 154.9, 152.3, 149.4, 148.6, 144.9, 137.4, 129.6, 128.4, 127.9, 114.4, 90.9, 84.8, 84.2, 81.7, 69.3, 39.4, 27.1, 25.3, 21.6. ESI-MS m/z: 490 [M+H]^+^.

**2’-*O*-Methyl-5’-*O*-tosyl-adenosine (11)**

White solid; yield: 27%.

^1^H NMR (400 MHz, CDCl_3_) δ 8.24 (s, 1H), 8.05 (s, 1H), 7.71 (d, *J* = 7.8 Hz, 2H), 7.23 (d, *J* = 7.7 Hz, 2H), 6.70 (s, 2H), 6.12 (s, 1H), 4.53 (d, *J* = 4.0 Hz, 1H), 4.44 – 4.21 (m, 4H), 3.48 (s, 3H), 2.34 (s, 3H). ^13^C NMR (101 MHz, CDCl_3_) δ 155.8, 153.1, 149.3, 145.3, 138.9, 132.2, 129.9, 127.9, 119.7, 86.8, 83.0, 81.8, 69.4, 68.7, 58.8, 21.6. ESI-MS m/z: 440 [M+H]^+^.

**2’,3’-*O*-Isopropylidene-5’-methylenediphosphate adenosine (CH_2_ADP, 3a)**

Light yellow foam solid; yield: 67%.

^1^H NMR (400 MHz, D_2_O) δ 8.27 (s, 1H), 8.01 (s, 1H), 6.08 (d, *J* = 3.3 Hz, 1H), 5.28 (dd, *J* = 6.2, 3.3 Hz, 1H), 5.10 (dd, *J* = 6.2, 2.4 Hz, 1H), 4.53 (d, *J* = 2.4 Hz, 1H), 4.05 – 3.90 (m, 2H), 1.99 (td, *J* = 19.8, 2.8 Hz, 2H), 1.58 (s, 3H), 1.36 (s, 3H). ^31^P NMR (162 MHz, D_2_O) δ 19.2 (d, *J* = 9.0 Hz), 15.4 (d, J = 9.1 Hz). ^13^C NMR (101 MHz, D_2_O) δ 155.3, 152.7, 148.4, 140.0, 118.4, 115.0, 90.0, 85.0, 84.9, 83.8, 81.3, 64.0, 26.1, 24.4. ESI-MS m/z: 464 [M-H]^-^.

**2’,3’-*O*-Isopropylidene-5’-methylenediphosphate-2-chloroadenosine (2-Cl-CH_2_ADP, 3b)**

Light yellow foam solid; yield: 63%.

^1^H NMR (400 MHz, D_2_O) δ 8.29 (s, 1H), 6.07 (s, 1H), 5.32 (dd, *J* = 5.9, 2.9 Hz, 1H), 5.20 – 5.09 (m, 1H), 4.55 (s, 1H), 3.99 (d, *J* = 4.0 Hz, 2H), 2.00 (td, *J* = 19.8, 2.4 Hz, 2H), 1.60 (s, 3H), 1.38 (s, 3H). ^31^P NMR (162 MHz, D_2_O) δ 18.9 (d, *J* = 8.9 Hz), 15.5 (d, *J* = 9.0 Hz). ^13^C NMR (101 MHz, D_2_O) δ 156.1, 153.7, 149.7, 140.2, 117.5, 114.9, 90.0, 85.3, 85.1, 83.9, 81.3, 64.1, 26.1, 24.4. ESI-MS m/z: 498 [M-H]^-^.

**2’,3’-*O*-Isopropylidene-5’-methylenediphosphate-2-bromoadenosine (2-Br-CH_2_ADP, 3c)**

Light yellow foam solid; yield: 70%.

^1^H NMR (400 MHz, D_2_O) δ 8.21 (s, 1H), 6.01 (s, 1H), 5.27 (dd, *J* = 5.6, 2.7 Hz, 1H), 5.08 (d, *J* = 6.1 Hz, 1H), 4.49 (s, 1H), 4.00 – 3.89 (m, 2H), 1.96 (dd, *J* = 28.8, 10.9 Hz, 2H), 1.55 (s, 3H), 1.34 (s, 3H). ^31^P NMR (162 MHz, D_2_O) δ 18.7 (td, *J* = 11.4, 5.9 Hz), 14.2 (td, *J* = 19.8, 9.0 Hz). ^13^C NMR (101 MHz, D_2_O) δ 155.7, 149.4, 144.4, 140.0, 117.6, 114.9, 89.8, 85.2, 85.1, 83.9, 81.3, 64.0, 26.1, 24.4. ESI-MS m/z: 542 [M-H]^-^.

**2’,3’-*O*-Isopropylidene-5’-methylenediphosphate-2-methoxyadenosine (2-OMe-CH_2_ADP, 3d)**

Light yellow foam solid; yield: 60%.

^1^H NMR (400 MHz, D_2_O) δ 8.01 (s, 1H), 6.04 (s, 1H), 5.41 – 5.33 (m, 1H), 5.17 – 5.04 (m, 1H), 4.45 (s, 1H), 3.91 (t, *J* = 4.9 Hz, 2H), 3.80 (s, 3H), 1.94 (t, 19.4Hz, 2H), 1.55 (s, 3H), 1.35 (s, 3H). ^31^P NMR (162 MHz, D_2_O) δ 18.2 (m), 14.8 (td, *J* = 19.9, 9.0 Hz). ^13^C NMR (101 MHz, D_2_O) δ 162.1, 156.3, 150.3, 139.2, 116.7, 114.8, 89.6, 85.3, 85.2, 83.5, 81.3, 64.0, 54.8, 26.1, 24.4. ESI-MS m/z: 496 [M+H]^+^.

**2’,3’-*O*-Isopropylidene-5’-methylenediphosphate-2-aminoadenosine (2-NH_2_-CH_2_ADP, 3e)**

Light yellow foam solid; yield: 52%.

^1^H NMR (400 MHz, D_2_O) δ 8.02 (s, 1H), 6.01 (d, *J* = 3.2 Hz, 1H), 5.30 (dd, *J* = 6.2, 3.3 Hz, 1H), 5.12 (dd, *J* = 6.2, 2.4 Hz, 1H), 4.49 (d, *J* = 2.7 Hz, 1H), 4.06 – 3.89 (m, 2H), 1.99 (td, *J* = 19.8, 1.1 Hz, 2H), 1.56 (s, 3H), 1.35 (s, 3H). ^31^P NMR (162 MHz, D_2_O) δ 18.3 (m), 14.7 (td, *J* = 19.7, 9.5 Hz). ^13^C NMR (101 MHz, D_2_O) δ 157.8, 154.1, 150.8, 138.2, 134.7, 114.8, 89.7, 85.0, 84.9, 83.7, 81.2, 64.0, 22.9, 21.2. ESI-MS m/z: 479 [M-H]^-^.

**2’,3’-*O*-Isopropylidene-5’-methylenediphosphate-6-methylaminoadenosine (6-NHCH_3_-CH_2_ADP, 3f)**

Light yellow foam solid; yield: 60%.

^1^H NMR (400 MHz, D_2_O) δ 8.20 (s, 1H), 7.97 (s, 1H), 6.07 – 5.98 (m, 1H), 5.25 (dd, *J* = 5.8, 3.5 Hz, 1H), 5.08 (dd, *J* = 6.2, 2.1 Hz, 1H), 4.50 (s, 1H), 4.05 – 3.87 (m, 2H), 2.89 (s, 3H), 1.95 (td, *J* = 19.8, 2.4 Hz, 2H), 1.57 (s, 3H), 1.35 (s, 3H). ^31^P NMR (162 MHz, D_2_O) δ 18.6 (td, *J* = 11.2, 5.7 Hz), 14.3 (td, *J* = 19.8, 9.0 Hz). ^13^C NMR (101 MHz, D_2_O) δ 154.7, 152.6, 147.2, 139.1, 118.6, 115.0, 89.8, 84.9, 84.7, 83.8, 81.3, 64.0, 58.0, 26.2, 24.4. ESI-MS m/z: 480 [M+H]^+^.

**2’,3’-*O*-Isopropylidene-5’-methylenediphosphate-6-dimethylaminoadenosine (6-N(CH_3_)_2_-CH_2_ADP, 3g)**

Light yellow foam solid; yield: 56%.

^1^H NMR (400 MHz, D_2_O) δ 8.03 (s, 1H), 7.71 (s, 1H), 5.90 (d, *J* = 3.3 Hz, 1H), 5.15 (dd, *J* = 6.2, 3.3 Hz, 1H), 4.99 (dd, *J* = 6.2, 2.4 Hz, 1H), 4.41 (d, *J* = 2.4 Hz, 1H), 3.95 – 3.77 (m, 2H), 2.99 (s, 6H), 1.91 (td, *J* = 19.8, 2.6 Hz, 2H), 1.51 (s, 3H), 1.29 (s, 3H). ^31^P NMR (162 MHz, D_2_O) δ 18.2 (ddd, *J* = 19.6, 12.7, 7.0 Hz), 14.6 (td, *J* = 19.9, 8.9 Hz). ^13^C NMR (101 MHz, D_2_O) δ 153.6, 151.7, 148. 5, 137.5, 118.4, 114.9, 89.5, 84.6, 84.5, 83.7, 81.2, 63.9, 38.7, 26.2, 24.4. ESI-MS m/z: 494 [M+H]^+^.

**2’-*O*-Methyl-5’-methylenediphosphate adenosine (2’-OMe-CH_2_ADP, 12)**

Light yellow foam solid; yield: 50%.

^1^H NMR (400 MHz, D_2_O) δ 8.35 (s, 1H), 8.00 (s, 1H), 6.00 (d, *J* = 5.5 Hz, 1H), 4.58 – 4.52 (m, 1H), 4.31 (t, *J* = 5.3 Hz, 1H), 4.23 (d, *J* = 3.0 Hz, 1H), 4.04 (dd, *J* = 5.0, 3.6 Hz, 2H), 3.35 (s, 3H), 2.04 (t, *J* = 19.7 Hz, 2H). ^31^P NMR (162 MHz, D_2_O) δ 19.2 (s), 14.1 (td, *J* = 19.6, 9.5 Hz). ^13^C NMR (101 MHz, D_2_O) δ 155.3, 152.7, 148.7, 139.8, 118.4, 85.3, 84.3, 84.2, 83.0, 68.9, 63.4, 58.2. ESI-MS m/z: 440 [M+H]^+^.

**2’,3’-*O*-Isopropylidene-5’-difluoromethylenediphosphate adenosine (CF_2_ADP** **3h)**

Light yellow foam solid; yield: 67%.

^1^H NMR (400 MHz, D_2_O) δ 8.35 (s, 1H), 8.12 (s, 1H), 6.16 (d, *J* = 3.4 Hz, 1H), 5.29 (dd, *J* = 6.1, 3.4 Hz, 1H), 5.14 (dd, *J* = 6.1, 2.1 Hz, 1H), 4.55 (s, 1H), 4.15 (dd, *J* = 9.2, 4.9 Hz, 2H), 1.59 (s, 3H), 1.36 (s, 3H). ^19^F NMR (376 MHz, D_2_O) δ -118.5 (dd, *J* = 84.5, 78.0 Hz). ^31^P NMR (162 MHz, D_2_O) δ 5.4 (td, *J* = 86.0, 53.3 Hz), 3.20 (td, *J* = 75.7, 53.1 Hz). ^13^C NMR (101 MHz, D_2_O) δ 155.1, 152.4, 148.3, 139.9, 118.3, 115.0, 89.7, 84.7, 84.6, 83.8, 81.1, 65.8, 26.1, 24.3. ESI-MS m/z: 502 [M+H]^+^.

**2’,3’-*O*-Isopropylidene-5’-difluoromethylenediphosphate-2-chloroadenosine (2-Cl-CF_2_ADP 3i)**

Light yellow foam solid; yield: 68%.

^1^H NMR (400 MHz, D_2_O) δ 8.36 (s, 1H), 6.11 (s, 1H), 5.30 (dd, *J* = 6.2, 3.4 Hz, 1H), 5.15 (d, *J* = 6.2 Hz, 1H), 4.56 (s, 1H), 4.24 – 4.07 (m, 2H), 1.59 (s, 3H), 1.37 (s, 3H). ^19^F NMR (376 MHz, D_2_O) δ -117.5 (t, *J* = 80.8 Hz). ^31^P NMR (162 MHz, D_2_O) δ 5.9 (td, *J*= 74.4, 34.0), 3.36 (td, *J* = 74.4, 52.8 Hz). ^13^C NMR (101 MHz, D_2_O) δ 158.0, 153.7, 149.7, 140.2, 117.7, 115.0, 89.8, 85.0, 84.9, 83.9, 81.1, 66.0, 26.1, 24.4. ESI-MS m/z: 536 [M+H]^+^.

**2’,3’-*O*-Isopropylidene-5’-difluoromethylenediphosphate-2-methoxyadenosine (2-OMe-CF_2_ADP 3j)**

Light yellow foam solid; yield: 58%.

^1^H NMR (400 MHz, D_2_O) δ 8.10 (s, 1H), 6.13 (d, *J* = 2.9 Hz, 1H), 5.42 (dd, *J* = 6.2, 2.9 Hz, 1H), 5.16 (dd, *J* = 6.2, 2.7 Hz, 1H), 4.50 (dd, *J* = 7.3, 4.6 Hz, 1H), 4.12 (t, *J* = 5.3 Hz, 2H), 3.87 (s, 3H), 1.58 (s, 3H), 1.37 (s, 3H). ^19^F NMR (376 MHz, D_2_O) δ -118.6 (dd, *J* = 83.4, 79.6 Hz). ^31^P NMR (162 MHz, D_2_O) δ 5.1 (td, *J* = 85.7, 54.8 Hz), 3.2 (td, *J* = 77.5, 53.9 Hz). ^13^C NMR (101 MHz, D_2_O) δ 161.9, 155.9, 150.0, 139.0, 114.8, 114.5, 89.3, 85.1, 85.1, 83.4, 81.1, 65.8, 54.7, 26.2, 24.4. ESI-MS m/z: 530 [M-H]^-^.

**2’,3’-*O*-Isopropylidene-5’-difluoromethylenediphosphate-2-aminoadenosine (2-NH_2_-CF_2_ADP 3k)**

Light yellow foam solid; yield: 60%.

^1^H NMR (400 MHz, D_2_O) δ 7.90 (s, 1H), 5.87 (d, *J* = 3.1 Hz, 1H), 5.16 – 5.13 (m, 1H), 5.04 (dd, *J* = 6.2, 2.6 Hz, 1H), 4.42 (d, *J* = 2.6 Hz, 1H), 4.11 (dd, *J* = 8.2, 5.0 Hz, 2H), 1.51 (s, 3H), 1.30 (s, 3H). ^19^F NMR (376 MHz, D_2_O) δ -118.2 (dd, *J* = 86.0, 75.0 Hz). ^31^P NMR (162 MHz, D_2_O) δ 5.7 (td, *J* = 86.4, 52.5 Hz), 3.4 (td, *J* = 74.8, 52.3 Hz). ^13^C NMR (101 MHz, D_2_O) δ 158.2, 154.2, 150.7, 137.8, 114.9, 112.3, 89.2, 84.7, 84.7, 83.7, 81.0, 65.8, 26.1, 24.4. ESI-MS m/z: 515 [M-H]^-^.

**2’,3’-*O*-Isopropylidene-5’-diphosphate-2-chloroadenosine (2-Cl-ADP 3l)**

Light yellow foam solid; yield: 55%.

^1^H NMR (400 MHz, D_2_O) δ 8.17 (s, 1H), 5.94 (d, *J* = 3.2 Hz, 1H), 5.18 (dd, *J* = 5.9, 3.2 Hz, 1H), 5.04 (dd, *J* = 6.0, 1.9 Hz, 1H), 4.46 (s, 1H), 3.95 (m, 2H), 1.49 (s, 3H), 1.27 (s, 3H). ^31^P NMR (162 MHz, D_2_O) δ -8.3 (d, *J* = 20.9 Hz), -11.3 (d, *J* = 20.7 Hz). ^13^C NMR (101 MHz, D_2_O) δ 155.9, 153.6, 149.5, 140.0, 117.2, 114.8, 89.8, 84.7, 83.8, 81.2, 65.4, 26.1, 24.3. ESI-MS m/z: 502 [M+H]^+^.

**1’’-*O*-Methyl-2’’,2’,3’’,3’-*O*-isopropylidene-5’-methylenediphosphoribose adenosine (Protected CH_2_ADPR 7a)**

White foam solid (NH_4_^+^ salt); yield: 20%.

^1^H NMR (400 MHz, D_2_O) δ 8.39 (s, 1H), 8.11 (s, 1H), 6.14 (d, *J* = 3.3 Hz, 1H), 5.31 (dd, *J* = 6.1, 3.3 Hz, 1H), 5.13 (dd, *J* = 6.1, 2.1 Hz, 1H), 4.87 (s, 1H), 4.65 (d, *J* = 6.0 Hz, 1H), 4.56 (d, *J* = 1.8 Hz, 1H), 4.47 (d, *J* = 5.9 Hz, 1H), 4.15 (t, *J* = 7.2 Hz, 1H), 4.10 – 3.91 (m, 2H), 3.83 – 3.50 (m, 2H), 3.20 (s, 3H), 2.03 (t, *J* = 19.1 Hz, 2H), 1.59 (s, 3H), 1.37 (s, 3H), 1.32 (s, 3H), 1.18 (s, 3H). ^31^P NMR (162 MHz, D_2_O) δ 17.0 (m). ^13^C NMR (101 MHz, D_2_O) δ 154.9, 152.0, 148.6, 140.4, 118.5, 115.0, 112.9, 108.5, 90.1, 85.1, 85.1, 85.1, 85.0, 84.1, 84.0, 81.4, 81.1, 64.2, 54.8, 26.2, 25.2, 24.5, 23.6. HRMS (ESI^+^): Calcd for C_23_H_36_N_5_O_13_P_2_ [M+H]^+^: 652.1785; Found: 652.1786.

**1’’-*O*-Methyl-2’’,2’,3’’,3’-*O*-isopropylidene-5’-methylenediphosphoribose-2-chloroadenosine (Protected 2-Cl-CH_2_ADPR 7b)**

White foam solid (NH_4_^+^ salt); yield: 23%.

^1^H NMR (400 MHz, D_2_O) δ 8.32 (s, 1H), 6.05 (d, *J* = 3.0 Hz, 1H), 5.31 (dd, *J* = 6.1, 3.0 Hz, 1H), 5.11 (dd, *J* = 6.1, 2.2 Hz, 1H), 4.84 (s, 1H), 4.60 (d, *J* = 6.0 Hz, 1H), 4.53 (s, 1H), 4.44 (d, *J* = 6.0 Hz, 1H), 4.08 (t, *J* = 7.3 Hz, 1H), 4.05 – 3.89 (m, 2H), 3.72 – 3.50 (m, 2H), 3.19 (s, 3H), 2.01 (td, *J* = 19.9, 1.7 Hz, 2H), 1.59 (s, 3H), 1.38 (s, 3H), 1.32 (s, 3H), 1.17 (s, 3H). ^31^P NMR (162 MHz, D_2_O) δ 16.9 (m). ^13^C NMR (101 MHz, D_2_O) δ 156.0, 153.7, 149.7, 140.3, 117.4, 114.9, 112.9, 108.5, 89.8, 85.3, 85.2, 85.1, 85.0, 84.1, 83.9, 81.4, 81.0, 64.2, 54.8, 26.2, 25.2, 24.5, 23.6. HRMS (ESI^+^): Calcd for C_23_H_35_N_5_O_14_P_2_Cl [M+H]^+^: 686.1395; Found: 686.1394.

**1’’-*O*-Methyl-2’’,2’,3’’,3’-*O*-isopropylidene-5’-methylenediphosphoribose-2-bromoadenosine (Protected 2-Br-CH_2_ADPR 7c)**

White foam solid (NH_4_^+^ salt); yield: 5%.

^1^H NMR (400 MHz, D_2_O) δ 8.31 (s, 1H), 6.05 (d, *J* = 3.3 Hz, 1H), 5.30 (dd, *J* = 6.0, 3.3 Hz, 1H), 5.09 (dd, *J* = 6.0, 2.0 Hz, 1H), 4.81 (s, 1H), 4.66 – 4.46 (m, 2H), 4.39 (d, *J* = 5.9 Hz, 1H), 4.20 – 3.84 (m, 3H), 3.57 (ddt, *J* = 11.9, 8.6, 5.4 Hz, 2H), 3.16 (s, 3H), 1.98 (t, *J* = 19.8 Hz, 2H), 1.56 (s, 3H), 1.34 (s, 3H), 1.28 (s, 3H), 1.14 (s, 3H). ^31^P NMR (162 MHz, D_2_O) δ 16.8 (m). ^13^C NMR (101 MHz, D_2_O) δ 155.9, 149.7, 144.5, 140.1, 117.7, 114.8, 112.8, 108.4, 89.8, 85.2, 85.1, 85.0, 84.9, 84.1, 83.9, 84.1, 81.0, 64.2, 54.7, 26.1, 25.1, 24.4, 23.5. HRMS (ESI^+^): Calcd for C_23_H_35_N_5_O_13_P_2_Br [M+H]^+^:730.0890; Found: 730.0883.

**1’’-*O*-Methyl-2’’,2’,3’’,3’-*O*-isopropylidene-5’-methylenediphosphoribose-2-methoxyadenosine (Protected 2-OMe-CH_2_ADPR 7d)**

White foam solid (NH_4_^+^ salt); yield: 15%.

^1^H NMR (400 MHz, D_2_O) δ 8.20 (s, 1H), 6.14 (d, *J* = 2.8 Hz, 1H), 5.41 (dd, *J* = 6.1, 2.8 Hz), 5.16 (dd, *J* = 6.1, 2.5 Hz, 1H), 4.91 (s, 1H), 4.55 – 4.51 (m, 2H), 4.18 (t, *J* = 7.3 Hz, 1H), 4.05 (d, *J* = 5.0 Hz, 2H), 3.91 (s, 3H), 3.71 (ddt, *J* = 14.1, 10.4, 7.0 Hz, 2H), 3.24 (s, 3H), 2.04 (t, *J* = 19.7 Hz, 2H), 1.61 (s, 3H), 1.40 (s, 3H), 1.37 (s, 3H), 1.22 (s, 3H). ^31^P NMR (162 MHz, D_2_O) δ 17.9 (m). ^13^C NMR (101 MHz, D_2_O) δ 160.9, 155.2, 150.4, 139.6, 114.9, 114.6, 112.9, 108.5, 89.8, 85.4, 85.4, 85.2, 85.1, 84.2, 83.7, 81.3, 81.1, 64.3, 55.2, 54.8, 26.1, 25.2, 24.5, 23.6. HRMS (ESI^+^): Calcd for C_24_H_38_N_5_O_14_P_2_ [M+H]^+^: 682.1890; Found: 682.1880.

**1’’-*O*-Methyl-2’’,2’,3’’,3’-*O*-isopropylidene-5’-methylenediphosphoribose-2-aminoadenosine (Protected 2-NH_2_-CH_2_ADPR 7e)**

White foam solid (NH_4_^+^ salt); yield: 15%.

^1^H NMR (400 MHz, D_2_O) δ 7.99 (s, 1H), 5.91 (d, *J* = 3.2 Hz, 1H), 5.20 (dd, *J* = 6.1, 3.2 Hz, 1H), 5.07 (dd, *J* = 6.1, 2.3 Hz, 1H), 4.84 (s, 1H), 4.60 (d, *J* = 6.0 Hz, 1H), 4.45 (m, 2H), 4.13 (t, *J* = 7.3 Hz, 1H), 4.08 – 3.92 (m, 2H), 3.65 (ddt, *J* = 17.8, 10.3, 7.1 Hz, 2H), 3.16 (s, 3H), 2.01 (td, *J* = 19.8, 1.7 Hz, 1H), 1.52 (s, 3H), 1.31 (s, 3H), 1.28 (s, 3H), 1.12 (s, 3H). ^31^P NMR (162 MHz, D_2_O) δ 16.7 (m). ^13^C NMR (101 MHz, D_2_O) δ 157.9, 154.1, 150.8, 138.0, 114.80, 112.9, 112.3, 108.4, 89.6, 85.1, 85.0, 85.0, 84.9, 84.1, 83.8, 81.3, 81.0, 64.2, 54.7, 26.1, 25.1, 24.4, 23.5. HRMS (ESI^+^): Calcd for C_23_H_37_N_6_O_14_P_2_ [M+H]^+^: 667.1894; Found: 667.1899.

**1’’-*O*-Methyl-2’’,2’,3’’,3’-*O*-isopropylidene-5’-methylenediphosphoribose-6-methylaminoadenosine (Protected 6-NHCH_3_-CH_2_ADPR 7f)**

White foam solid (NH_4_^+^ salt); yield: 15%.

^1^H NMR (400 MHz, D_2_O) δ 8.26 (s, 1H), 8.00 (s, 1H), 6.05 (d, *J* = 3.3 Hz, 1H), 5.28 (dd, *J* = 6.1, 3.3 Hz, 1H), 5.10 (dd, *J* = 6.1, 2.1 Hz, 1H), 4.81 (s, 1H), 4.58 (d, *J* = 5.9 Hz, 1H), 4.52 (s, 1H), 4.40 (d, *J* = 5.9 Hz, 1H), 4.08 (t, *J* = 7.2 Hz, 1H), 4.04 – 3.93 (m, 2H), 3.60 (dtd, *J* = 14.1, 10.4, 7.0 Hz, 2H), 3.16 (s, 3H), 2.92 (s, 3H), 1.99 (td, *J* = 19.9, 2.8 Hz, 2H), 1.58 (s, 3H), 1.37 (s, 3H), 1.28 (s, 3H), 1.14 (s, 3H). ^31^P NMR (162 MHz, D_2_O) δ 16.9 (m). ^13^C NMR (101 MHz, D_2_O) δ 154.4, 152.1, 147.2, 139.4, 118.7, 114.9, 112.9, 108.4, 89.9, 85.1, 85.0, 85.0, 84.9, 84.1, 83.9, 81.4, 81.0, 64.2, 54.7, 46.6, 26.2, 25.2, 24.5, 23.6. HRMS (ESI^+^): Calcd for C_24_H_38_N_5_O_13_P_2_ [M+H]^+^: 666.1941; Found: 666.1946.

**1’’-*O*-Methyl-2’’,2’,3’’,3’-*O*-isopropylidene-5’-methylenediphosphoribose-6-dimethylaminoadenosine (Protected 6-N(CH_3_)_2_-CH_2_ADPR 7g)**

White foam solid (NH_4_^+^ salt); yield: 30%.

^1^H NMR (400 MHz, D_2_O) δ 8.26 (s, 1H), 7.93 (s, 1H), 6.06 (d, *J* = 3.4 Hz, 1H), 5.28 (dd, *J* = 6.0, 3.4 Hz, 1H), 5.10 (dd, *J* = 6.1, 2.0 Hz, 1H), 4.79 (s, 1H), 4.56 (d, *J* = 5.9 Hz, 1H), 4.52 (d, *J* = 1.7 Hz, 1H), 4.35 (d, *J* = 5.9 Hz, 1H), 4.12 – 3.92 (m, 3H), 3.70 – 3.47 (m, 2H), 3.21 (s, 6H), 3.14 (s, 3H), 1.99 (td, *J* = 19.9, 2.1 Hz, 2H), 1.59 (s, 3H), 1.37 (s, 3H), 1.26 (s, 3H), 1.12 (s, 3H). ^31^P NMR (162 MHz, D_2_O) δ 16.9 (d, *J* = 17.2 Hz). ^13^C NMR (101 MHz, D_2_O) δ 153.9, 151.7, 148.8, 138.0, 118.8, 114.9, 112.8, 108.5, 89.8, 85.1, 85.0, 84.9, 84.8, 84.0, 83.8, 81.4, 81.0, 64.2, 54.7, 46.6, 26.3, 25.2, 24.5, 23.6. HRMS (ESI^+^): Calcd for C_25_H_40_N_5_O_13_P_2_ [M+H]^+^: 680.2098; Found: 680.2089.

**1’’-*O*-Methyl-2’’,3’’-*O*-isopropylidene-2’-methoxy-5’-methylenediphosphoribose adenosine (Protected** **2’-OMe-CH_2_ADPR 13)**

White foam solid (NH_4_^+^ salt); yield: 12%.

^1^H NMR (400 MHz, D_2_O) δ 8.53 (d, *J* = 3.1 Hz, 1H), 8.19 (d, *J* = 6.3 Hz, 1H), 6.11 (dd, *J* = 5.1, 2.9 Hz, 1H), 4.90 (s, 1H), 4.66 (d, *J* = 6.0 Hz, 1H), 4.63 (t, *J* = 4.0 Hz, 1H), 4.50 (d, *J* = 6.0 Hz, 1H), 4.41 (d, *J* = 4.0 Hz, 1H), 4.30 (d, *J* = 2.6 Hz, 1H), 4.18 (t, *J* = 7.3 Hz, 1H), 4.13 (d, *J* = 3.9 Hz, 2H), 3.85 – 3.63 (m, 2H), 3.41 (s, 3H), 3..22 (s, 3H), 2.14 (t, *J* = 19.9 Hz, 2H), 1.29 (s, 3H), 1.16 (s, 3H). ^31^P NMR (162 MHz, D_2_O) δ 17.8 (m). ^13^C NMR (101 MHz, D_2_O) δ 155.0, 151.9, 148.8, 140.4, 118.6, 112.9, 108.5, 85.5, 85.1, 85.0, 84.5, 84.1, 83.2, 81.1, 68.9, 64.2, 63.6, 58.2, 54.8, 25.1, 23.5. HRMS (ESI^+^): Calcd for C_21_H_34_N_5_O_14_P_2_ [M+H]^+^: 626.1628; Found: 626.1622. **1’’-*O*-Methyl-2’’,2’,3’’,3’-*O*-isopropylidene-5’-difluoromethylenediphosphoribose adenosine (Protected CF_2_ADPR 7h)**

White foam solid (NH_4_^+^ salt); yield: 30%.

^1^H NMR (400 MHz, D_2_O) δ 8.43 (s, 1H), 8.21 (s, 1H), 6.20 (d, *J* = 3.3 Hz, 1H), 5.31 (dd, *J* = 6.0, 3.2 Hz, 1H), 5.15 (dd, *J* = 6.0, 1.5 Hz, 1H), 4.92 (s, 1H), 4.69 (d, *J* = 6.0Hz, 1H, partially hidden under HDO peak), 4.60 (s, 1H), 4.53 (d, *J* = 6.0 Hz, 1H), 4.19 (m, 3H), 3.87 (m, 2H), 3.24 (s, 3H), 1.60 (s, 3H), 1.38 (s, 3H), 1.34 (s, 3H), 1.20 (s, 3H). ^19^F NMR (376 MHz, D_2_O) δ -118.6 (td, *J* = 83.1, 7.3 Hz). ^31^P NMR (162 MHz, D_2_O) δ 3.3 (t, *J* = 83.5 Hz). ^13^C NMR (101 MHz, D_2_O) δ 153.6, 150.0, 148.5, 140.9, 118.6, 115.0, 112.9, 108.5, 90.4, 85.0, 85.0, 85.0, 84.9, 84.1, 84.1, 81.3, 80.9, 66.2, 54.8, 26.1, 25.2, 24.4, 23.5. HRMS (ESI^+^): Calcd for C_23_H_34_F_2_N_5_O_13_P_2_ [M+H]^+^: 688.1596; Found: 688.1605.

**1’’-*O*-Methyl-2’’,2’,3’’,3’-*O*-isopropylidene-5’-difluoromethylenediphosphoribose-2-chloroadenosine (Protected 2-Cl-CF_2_ADPR 7i)**

White foam solid (NH_4_^+^ salt); yield: 25%.

^1^H NMR (400 MHz, D_2_O) δ 8.36 (s, 1H), 6.12 (d, *J* = 3.4 Hz, 1H), 5.32 (dd, *J* = 6.0, 3.4 Hz, 1H), 5.14 (dd, *J* = 6.0, 1.9 Hz, 1H), 4.90 (s, 1H), 4.59 (d, *J* = 6.0 Hz, 1H), 4.48 (d, *J* = 6.0 Hz, 1H), 4.27 – 4.15 (m, 2H), 4.11 (t, *J* = 7.3 Hz, 1H), 3.91 – 3.68 (m, 2H), 3.24 (s, 3H), 1.61 (s, 3H), 1.39 (s, 3H), 1.34 (s, 3H), 1.19 (s, 3H). ^19^F NMR (376 MHz, D_2_O) δ -118.7 (m). ^31^P NMR (162 MHz, D_2_O) δ 3.3 (dd, *J* = 83.9, 59.5 Hz). ^13^C NMR (101 MHz, D_2_O) δ 156.0, 153.8, 149.8, 140.2, 117.4, 115.0, 112.9, 108.5, 89.7, 85.0, 84.9, 84.9, 84.9, 84.1, 83.9, 81.3, 80.9, 66.3, 54.8, 26.2, 25.2, 24.5, 23.5. HRMS (ESI^+^): Calcd for C_23_H_33_F_2_N_5_O_13_P_2_Cl [M+H]^+^: 722.1207; Found: 722.1201.

**1’’-*O*-Methyl-2’’,2’,3’’,3’-*O*-isopropylidene-5’-difluoromethylenediphosphoribose-2-methoxyadenosine (Protected 2-OMe-CF_2_ADPR 7j)**

White foam solid (NH_4_^+^ salt); yield: 23%.

^1^H NMR (400 MHz, D_2_O) δ 8.07 (s, 1H), 6.08 (d, *J* = 2.8 Hz, 1H), 5.37 (dd, *J* = 6.1, 2.8 Hz, 1H), 5.12 (dd, *J* = 6.1, 2.5 Hz, 1H), 4.89 (s, 1H), 4.65 (d, *J* = 5.9 Hz, 1H), 4.48 (d, *J* = 5.9 Hz, 1H), 4.17 (t, *J* = 7.2 Hz, 1H), 4.12 (s, 1H), 3.89 – 3.74 (m, 5H), 3.21 (s, 3H), 1.58 (s, 3H), 1.38 (s, 3H), 1.33 (s, 3H), 1.18 (s, 3H). ^19^F NMR (376 MHz, D_2_O) δ -118.7 (t, *J* = 82.8 Hz). ^31^P NMR (162 MHz, D_2_O) δ 3.3 (t, *J* = 83.1 Hz). ^13^C NMR (101 MHz, D_2_O) δ 161.6, 155.8, 150.3, 139.3, 114.9, 114.7, 112.9, 108.5, 89.6, 85.2, 85.2, 85.0, 84.9, 84.2, 83.6, 81.2, 80.9, 66.2, 54.9, 54.8, 26.2, 25.2, 24.5, 23.6. HRMS (ESI^+^): Calcd for C_24_H_36_F_2_N_5_O_14_P_2_ [M+H]^+^: 718.1702; Found: 718.1690.

**1’’-*O*-Methyl-2’’,2’,3’’,3’-*O*-isopropylidene-5’-difluoromethylenediphosphoribose-2-aminoadenosine (Protected 2-NH_2_-CF_2_ADPR 7k)**

White foam solid (NH_4_^+^ salt); yield: 17%.

^1^H NMR (400 MHz, D_2_O) δ 8.06 (s, 1H), 5.96 (d, *J* = 3.1 Hz, 1H), 5.21 (d, *J* = 6.1, 3.1 Hz, 1H), 5.13 (dd, *J* = 6.1, 2.2 Hz, 1H), 4.92 (s, 1H), 4.54 (d, *J* = 5.9 Hz, 2H), 4.55 – 4.50(m, 2H), 4.28 – 4.11 (m, 3H), 3.87 (m, 2H), 3.24 (s, 3H), 1.58 (s, 3H), 1.37 (s, 3H), 1.34 (s, 3H), 1.19 (s, 3H). ^19^F NMR (376 MHz, D_2_O) δ -118.7 (t, *J* = 85.4 Hz). ^31^P NMR (162 MHz, D_2_O) δ 3.3 (t, *J* = 84.1 Hz). ^13^C NMR (101 MHz, D_2_O) δ 154.8, 151.6, 151.0, 139.2, 114.9, 113.0, 111.6, 108.5, 90.3, 85.1, 85.0, 85.0, 85.0, 84.2, 84.1, 81.2, 81.0, 66.3, 54.8, 26.1, 25.2, 24.4, 23.6. HRMS (ESI^+^): Calcd for C_23_H_35_F_2_N_6_O_13_P_2_ [M+H]^+^: 703.1705; Found: 703.1696.

**1’’-*O*-Methyl-2’’,2’,3’’,3’-*O*-isopropylidene-5’-diphosphoribose-2-chloroadenosine (Protected 2-Cl-ADPR** **7l)**

White foam solid (NH_4_^+^ salt); yield: 18%.

^1^H NMR (400 MHz, D_2_O) δ 8.31 (s, 1H), 6.07 (d, *J* = 3.4 Hz, 1H), 5.26 (dd, *J* = 6.0, 3.4 Hz, 1H), 5.10 (dd, *J* = 6.1, 2.0 Hz, 1H), 4.81 (s, 1H), 4.61 – 4.50 (m, 2H), 4.42 (d, *J* = 5.9 Hz, 1H), 4.14 – 3.96 (m, 3H), 3.63 (dd, *J* = 12.3, 5.2 Hz, 2H), 3.14 (s, 3H), 1.55 (s, 3H), 1.33 (s, 3H), 1.29 (s, 3H), 1.14 (s, 3H). ^31^P NMR (162 MHz, D_2_O) δ -11.7 (m). ^13^C NMR (101 MHz, D_2_O) δ 156.0, 153.7, 149.8, 140.0, 117.4, 114.9, 112.9, 108.4, 89.7, 84.7, 84.6, 84.5, 84.1, 83.8, 81.2, 80.9, 65.8, 54.6, 26.1, 25.1, 24.4, 23.5. HRMS (ESI^+^): Calcd for C_22_H_33_N_5_O_14_P_2_ [M+H]^+^: 688.1188; Found: 688.1184.

**1’’-*O*-Methyl-2’’,3’’-*O*-isopropylidene-5’-methylenediphosphoribose adenosine (8a)**

White foam solid (NH_4_^+^ salt); yield: 32%.

^1^H NMR (400 MHz, D_2_O) δ 8.48 (s, 1H), 8.14 (s, 1H), 6.01 (d, *J* = 5.5 Hz, 1H), 4.85 (s, 1H), 4.66 (t, *J* = 5.5 Hz, 1H), 4.60 (d, *J* = 6.0 Hz, 1H), 4.45 – 4.41 (m, 2H), 4.26 (d, *J* = 6.0 Hz, 1H), 4.17 – 4.02 (m, 3H), 3.68 (ddd, *J* = 24.3, 14.1, 7.2 Hz, 2H), 3.18 (s, 3H), 2.09 (t, *J* = 20.0 Hz, 2H), 1.24 (s, 3H), 1.11 (s, 3H). ^31^P NMR (162 MHz, D_2_O) δ 17.0 (m). ^13^C NMR (101 MHz, D_2_O) δ 156.3, 153.8, 150.2, 140.1, 117.7, 112.8, 108.5, 87.1, 85.0, 84.9, 84.1, 83.9, 81.0, 74.3, 70.2, 64.2, 63.6, 54.7, 25.1, 23.5. HRMS (ESI^+^): Calcd for C_20_H_32_N_5_O_13_P_2_ [M+H]^+^: 612.1472; Found: 612.1462.

**Methylenediphosphoribose adenosine (CH_2_ADPR 10a)**

White foam solid (Et_3_N^+^ salt); yield: 25%.

^1^H NMR (400 MHz, D_2_O) δ 8.49 (s, 1H), 8.18 (s, 1H), 6.06 (d, *J* = 5.6 Hz, 1H), 5.20 (5.27, d, *J* = 4.1 Hz, 0.4H, 5.14, d, *J* = 2.1 Hz, 0.6H), 4.73 (s, 1H, partially hidden under HDO peak), 4.56 – 4.46 (m, 1H), 4.32 (d, *J* = 3.1 Hz, 1H), 4.29 – 4.23 (m, 1H), 4.17 – 4.09 (m, 3H), 4.05 – 3.88 (m, 3H), 2.16 (ddd, *J* = 27.5, 14.3, 5.0 Hz, 2H). ^31^P NMR (162 MHz, D_2_O) δ 18.1 (m). ^13^C NMR (101 MHz, D_2_O) δ 154.8, 151.8, 149.0, 140.4, 118.6, 101.3, 96.4, 87.1, 84.2, 82.0, 75.3, 74.3, 70.7, 70.4, 64.6, 63.6. HRMS (ESI^+^): Calcd for C_16_H_26_N_5_O_13_P_2_ [M+H]^+^: 558.1001; Found: 558.1001.

**1’’-*O*-Methyl-2’’,3’’-*O*-isopropylidene-5’-methylenediphosphoribose-2-chloroadenosine (8b)**

White foam solid (NH_4_^+^ salt); yield: 32%.

^1^H NMR (400 MHz, D_2_O) δ 8.41 (s, 1H), 5.91 (d, *J* = 5.3 Hz, 1H), 4.84 (s, 1H, partly hidden under HDO peak), 4.64 (t, *J* = 5.2 Hz, 1H), 4.57 (d, *J* = 6.0 Hz, 1H), 4.44 (d, *J* = 6.0 Hz, 1H), .4.41 (t, *J* = 4.4 Hz, 1H) 4.25 (d, *J* = 2.5 Hz, 1H), 4.16 – 3.98 (m, 3H), 3.72 –3.58 (m, 2H), 3.18 (s, 3H), 2.08 (t, *J* = 19.9 Hz, 2H), 1.26 (s, 3H), 1.12 (s, 3H). ^31^P NMR (162 MHz, D_2_O) δ 17.7 (m). ^13^C NMR (101 MHz, D_2_O) δ 156.3, 153.8, 150.2, 140.1, 117.7, 112.8, 108.5, 87.1, 85.0, 84.9, 84.1, 83.8, 81.0, 74.3, 70.2, 64.2, 63.6, 54.7, 25.1, 23.5. HRMS (ESI^+^): Calcd for C_20_H_31_N_5_O_13_P_2_Cl [M+H]^+^: 646.1082; Found: 646.1081.

**Methylenediphosphoribose-2-chloroadenosine (2-Cl-CH_2_ADPR 10b)**

White foam solid (Et_3_N^+^ salt); yield: 22%.

^1^H NMR (400 MHz, D_2_O) δ 8.38 (s, 1H), 5.90 (d, *J* = 5.3 Hz, 1H), 5.17(5.24, d, *J* = 4.0 Hz, 0.4H, 5.10 s, 0.6H), 4.65 (t, *J* = 5.0 Hz, 1H), 4.44 (d, *J* = 4.1 Hz, 1H), 4.27 (s, 1H), 4.22 (t, *J* = 5.2 Hz, 1H), 4.07 (s, 3H), 3.99 – 3.72 (m, 3H), 2.11 (td, *J* = 19.9, 7.7 Hz, 2H). ^31^P NMR (162 MHz, D_2_O) δ 18.1 (dd, *J* = 20.5, 9.2 Hz). ^13^C NMR (101 MHz, D_2_O) δ 156.0, 153.7, 149.0, 141.1, 117.4, 101.2, 96.3, 87.1, 84.0, 82.0, 75.2, 74.3, 70.6, 70.2, 64.5, 63.5. HRMS (ESI^+^): Calcd for C_16_H_25_N_5_O_13_P_2_Cl [M+H]^+^: 592.0613; Found: 592.0619.

**Methylenediphosphoribose-2-bromoadenosine (2-Br-CH_2_ADPR 10c)**

White foam solid (Et_3_N^+^ salt); yield: 39%.

^1^H NMR (400 MHz, D_2_O) δ 8.38 (s, 1H), 5.93 (d, *J* = 5.4 Hz, 1H), 5.17 (5.24, d, *J* = 4.1 Hz, 0.4H, 5.11 ,d, *J* = 2.1 Hz, 0.6H), 4.66 (t, *J* = 5.2 Hz, 1H), 4.45 (t, *J* = 4.5 Hz, 1H), 4.28 (d, *J* = 3.0 Hz, 1H), 4.23 (t, *J* = 5.2 Hz, 1H), 4.12 – 4.02 (m, 3H), 3.99 – 3.85 (m, 3H), 2.11 (td, *J* = 19.8, 8.3 Hz, 2H). ^31^P NMR (162 MHz, D_2_O) δ 18.1 (s). ^13^C NMR (101 MHz, D_2_O) δ 155.9, 150.0, 144.5, 139.9, 117.9, 101.2, 96.3, 87.1, 84.0, 82.0, 75.2, 74.3, 70.6, 70.2, 64.4, 63.5. HRMS (ESI^+^): Calcd for C_16_H_25_N_5_O_13_P_2_ [M+H]^+^: 636.0107; Found: 636.0103.

**Methylenediphosphoribose-2-methoxyadenosine (2-OMe-CH_2_ADPR 10d)**

White foam solid (Et_3_N^+^ salt); yield: 40%.

^1^H NMR (400 MHz, D_2_O) δ 8.26 (s, 1H), 5.99 (d, *J* = 5.3 Hz, 1H), 5.22 (5.28, d, *J* = 4.1 Hz, 0.4H, 5.15, d, *J* = 2.1 Hz, 0.6H), 4.78 (t, *J* = 5.2 Hz, 1H), 4.53 (t, *J* = 4.7 Hz, 1H), 4.33 – 4.22 (m, 2H), 4.18 – 4.04 (m, 3H), 4.03 – 3.85 (m, 3H), 3.93 (s, 3H), 2.14 (td, *J* = 19.9, 8.1 Hz, 2H). ^31^P NMR (162 MHz, D_2_O) δ 18.1 (dd, *J* = 14.4, 6.5 Hz). ^13^C NMR (101 MHz, D_2_O) δ 161.0, 155.3, 150.8, 139.5, 114.6, 101.3, 96.4, 87.4, 83.7, 82.0, 75.3, 73.8, 70.6, 70.3, 64.5, 63.7, 55.3. HRMS (ESI^+^): Calcd for C_17_H_28_N_5_O_14_P_2_ [M+H]^+^: 588.1108; Found: 588.1102.

**Methylenediphosphoribose-2-aminoadenosine (2-NH_2_-CH_2_ADPR 10e)**

White foam solid (Et_3_N^+^ salt); yield: 35%.

^1^H NMR (400 MHz, D_2_O) δ 8.13 (s, 1H), 5.82 (d, *J* = 5.5 Hz, 1H), 5.20 (5.26, d, *J* = 4.1 Hz, 0.4H, 5.13, d, *J* = 2.1 Hz, 0.6H), 4.62 (m, 2H), 4.50 –4.39 (m, 2H), 4.30 – 3.84 (m, 6H) , 2.31 – 2.04 (m, 2H). ^31^P NMR (162 MHz, D_2_O) δ 17.5 (s). ^13^C NMR (101 MHz, D_2_O) δ 160.1, 156.1, 151.2, 137.3, 126.4, 101.2, 96.8, 86.6, 83.6, 81.8, 75.2, 73.7, 70.5, 70.3, 64.7, 63.6. HRMS (ESI^+^): Calcd for C_16_H_27_N_6_O_13_P_2_ [M+H]^+^: 573.1111; Found: 573.1132.

**Methylenediphosphoribose-6-methylaminoadenosine (6-NHCH_3_-CH_2_ADPR 10f)**

White foam solid (Et_3_N^+^ salt); yield: 33%.

^1^H NMR (400 MHz, D_2_O) δ 8.38 (s, 1H), 8.07 (s, 1H), 5.98 (d, *J* = 5.6 Hz, 1H), 5.17 (5.23, d, *J* = 4.1 Hz, 0.4H, 5.11, d, *J* = 2.0 Hz, 0.6H), 4.68 (1H, partially hidden under HDO peak), 4.47 – 4.43 (m), 4.30 – 4.20 (m, 2H), 4.15 – 3.97 (m, 3H), 4.00 – 3.83 (m, 3H), 2.96 (s, 3H), 2.12 (ddd, *J* = 24.0, 13.9, 6.1 Hz, 2H). ^31^P NMR (162 MHz, D_2_O) δ 18.1 (s). ^13^C NMR (101 MHz, D_2_O) δ 154.4, 152.2, 139.4, 118.7, 105.0, 101.2, 96.3, 86.9, 84.0, 82.0, 75.2, 74.2, 70.6, 70.3, 64.5, 63.6, 46.8. HRMS (ESI^+^): Calcd for C_17_H_28_N_5_O_13_P_2_ [M+H]^+^: 572.1159; Found: 572.1152.

**Methylenediphosphoribose-6-dimethylaminoadenosine (6-N(CH_3_)_2_-CH_2_ADPR 10g)**

White foam solid (Et_3_N^+^ salt); yield: 36%.

^1^H NMR (400 MHz, D_2_O) δ 8.31 (s, 1H), 7.95 (s, 1H), 5.95 (d, *J* = 5.5 Hz, 1H), 5.14 (5.20, d, *J* = 4.1 Hz, 0.4H, 5.08, d, *J* = 2.0 Hz, 0.6H), 4.68 – 4.62 (m, 1H), 4.46 – 4.40 (m, 1H), 4.27 – 4.17 (m, 2H), 4. 09 – 4.06(m, 3H), 3.96 – 3.80 (m, 3H), 3.21 (s, 6H), 2.10 (td, *J* = 19.9, 7.8 Hz, 2H). ^31^P NMR (162 MHz, D_2_O) δ 18.1 (s). ^13^C NMR (101 MHz, D_2_O) δ 154.4, 152.2, 139.4, 118.7, 105.0, 101.2, 96.3, 86.9, 84.0, 82.0, 75.2, 74.2, 70.6, 70.3, 64.5, 63.6, 46.7. HRMS (ESI^+^): Calcd for C_18_H_30_N_5_O_13_P_2_ [M+H]^+^: 586.1315; Found: 586.1326.

**Methylenediphosphoribose-2’-methoxyadenosine (2’-OMe-CH_2_ADPR 14)**

White foam solid (Et_3_N^+^ salt); yield: 45%.

^1^H NMR (400 MHz, D_2_O) δ 8.47 (s, 1H), 8.13 (s, 1H), 6.09 (d, *J* = 5.8 Hz, 1H), 5.14 (5.25, d, *J* = 4.1 Hz, 0.4H, 5.12, d, *J* = 2.0 Hz, 0.6H), 4.67 – 4.50 (m, 1H), 4.43 (t, *J* = 5.3 Hz, 1H), 4.34 – 4.20 (m, 2H), 4.20 – 4.02 (m, 3H), 4.02 – 3.83 (m, 3H), 3.41 (s, 3H), 2.13 (td, *J* = 19.9, 8.4 Hz, 2H). ^31^P NMR (162 MHz, D_2_O) δ 18.0 (m). ^13^C NMR (101 MHz, D_2_O) δ 155.5, 152.8, 148.9, 140.1, 118.5, 101.3, 96.4, 85.3, 84.6, 83.0, 82.0, 75.3, 70.6, 69.0, 64.5, 63.6, 58.2. HRMS (ESI^+^): Calcd for C_17_H_28_N_5_O_13_P_2_ [M+H]^+^: 572.1159; Found: 572.1169.

**Difluoromethylenediphosphoribose adenosine (CF_2_ADPR 10h)**

White foam solid (Et_3_N^+^ salt); yield: 39%.

^1^H NMR (400 MHz, D_2_O) δ 8.50 (s, 1H), 8.23 (s, 1H), 6.20 (d, *J* = 5.9 Hz, 1H), 5.23 (5.30, d, *J* = 4.0 Hz, 0.4H, 5.17, d, *J* = 2.0 Hz, 0.6H), 4.72 (1H, partially hidden under HDO peak), 4.53 – 4.46 (m, 1H), 4.33 (s, 1H), 4.30 – 4.20 (m, 3H), 4.20 – 3.92 (m, 4H). ^19^F NMR (376 MHz, D_2_O) δ -118.7 (dd, *J* = 102.3, 63.1 Hz). ^31^P NMR (162 MHz, D_2_O) δ 3.5 (t, *J* = 82.2 Hz). ^13^C NMR (101 MHz, D_2_O) δ 155.3, 152.5, 149.0, 139.9, 118.5, 101.2, 96.3, 86.7, 84.0, 81.6, 75.1, 74.3, 70.5, 70.4, 66.6, 65.5. HRMS (ESI^+^): Calcd for C_16_H_24_F_2_N_5_O_13_P_2_ [M+H]^+^: 594.0814; Found: 594.0792.

**Difluoromethylenediphosphoribose-2-chloroadenosine (2-Cl-CF_2_ADPR 10i)**

White foam solid (NH_4_^+^ salt); yield: 32%.

^1^H NMR (400 MHz, D_2_O) δ 8.41 (s, 1H), 5.97 (d, *J* = 5.7 Hz, 1H), 5.22 (5.29, d, *J* = 4.1 Hz, 0.4H, 5.14, d, *J* = 2.2 Hz, 0.6H), 4.65 (t, *J* = 5.4 Hz, 1H), 4.50 – 4.43 (m, 1H), 4.31 (d, *J* = 2.4 Hz, 1H), 4.28 – 4.20 (m, 3H), 4.19 – 3.89 (m, 4H). ^19^F NMR (376 MHz, D_2_O) δ -118.8 (td, *J* = 82.3, 35.3 Hz). ^31^P NMR (162 MHz, D_2_O) δ 3.5 (t, *J* = 81.4 Hz). ^13^C NMR (101 MHz, D_2_O) δ 156.2, 153.8, 150.2, 134.0, 117.6, 101.2, 96.3, 86.9, 84.0, 81.7, 75.1, 74.4, 70.4, 70.2, 66.6, 65.5. HRMS (ESI^+^): Calcd for C_16_H_23_F_2_N_5_O_13_P_2_Cl [M+H]^+^: 628.0424; Found: 628.0441.

**1’’-*O*-Methyl-2’’,3’’-*O*-isopropylidene-5’-difluoromethylenediphosphoribose-2-chloroadenosine (8c)**

White foam solid (NH_4_^+^ salt); yield: 29%.

^1^H NMR (400 MHz, D_2_O) δ 8.36 (s, 1H), 5.92 (d, *J* = 5.7 Hz, 1H), 4.86 (s, 1H), 4.67 – 4.60 (m, 1H), 4.56 (d, *J* = 6.0 Hz, 1H), 4.46 (d, *J* = 5.9 Hz, 1H), 4.40 (dd, *J* = 5.0, 3.8 Hz, 1H), 4.26 (d, *J* = 1.7 Hz, 1H), 4.23 – 4.16 (m, 2H), 4.10 (t, *J* = 7.3 Hz, 1H), 3.93 – 3.72 (m, 2H), 3.19 (s, 3H), 1.27 (s, 3H), 1.13 (s, 3H). ^19^F NMR (376 MHz, D_2_O) δ -118.7 (t, *J* = 83.3 Hz). ^31^P NMR (162 MHz, D_2_O) δ 4.1 (td, *J* = 83.1, 38.4 Hz). ^13^C NMR (101 MHz, D_2_O) δ 156.2, 153.8, 150.2, 140.0, 117.6, 112.9, 108.5, 86.9, 84.8, 84.8, 84.1, 84.1, 80.9, 74.4, 70.4, 66.2, 65.6, 54.8, 25.1, 23.4. HRMS (ESI^+^): Calcd for C_20_H_29_F_2_N_5_O_13_P_2_Cl [M+H]^+^: 682.0894; Found: 682.0886.

**Difluoromethylenediphosphoribose-2-methoxyadenosine (2-OMe-CF_2_ADPR 10j)**

White foam solid (Et_3_N^+^ salt); yield: 35%.

^1^H NMR (400 MHz, D_2_O) δ 8.17 (s, 1H), 5.95 (d, *J* = 5.7 Hz, 1H), 5.19, (5.26, d, *J* = 4.1 Hz, 0.4H, 5.12, d, *J* = 2.0 Hz, 0.6H), 4.79 – 4.73 (m, 1H), 4.47 (dd, *J* = 8.8, 4.7 Hz, 1H), 4.29 – 4.15 (m, 4H), 4.13 – 3.89 (m, 4H), 3.84 (s, 3H). ^31^P NMR (162 MHz, D_2_O) δ 4.3 (t, *J* = 81.1 Hz). ^19^F NMR (376 MHz, D_2_O) δ -118.5 (td, *J* = 82.8, 42.1 Hz). ^13^C NMR (101 MHz, D_2_O) δ 162.2, 156.3, 151.1, 139.2, 114.9, 101.2, 96.4, 87.1, 83.8, 81.7, 75.2, 73.7, 70.5, 70.5, 66.6, 65.7, 55.0, . HRMS (ESI^+^): Calcd for C_17_H_26_F_2_N_5_O_14_P_2_ [M+H]^+^: 624.0920; Found: 624.0937.

**Difluoromethylenediphosphoribose-2-aminoadenosine (2-NH_2_-CF_2_ADPR 10k)**

White foam solid (Et_3_N^+^ salt); yield: 39%.

^1^H NMR (400 MHz, D_2_O) δ 8.18 (s, 1H), 5.90 (d, *J* = 6.1 Hz, 1H), 5.24 (5.31, d, *J* = 4.2 Hz, 0.4H, 5.16, d, *J* = 2.3 Hz, 0.6H), 4.65 (1H, partially hidden under HDO peak), 4.53 – 4.44 (m, 1H), 4.29 (d, *J* = 6.3 Hz, 1H), 4.26 (d, *J* = 4.4 Hz, 3H), 4.20 – 3.96 (m, 4H). ^19^F NMR (376 MHz, D_2_O) δ -118.8 (td, *J* = 82.4, 38.2 Hz). ^31^P NMR (162 MHz, D_2_O) δ 4.3 (ddd, *J* = 86.0, 80.9, 8.5 Hz). ^13^C NMR (101 MHz, D_2_O) δ 158.7, 154.9, 151.2, 137.9, 112.6, 101.2, 96.4, 86.5, 83.8, 81.7,75.2, 73.9, 70.5, 70.4, 66.7, 65.6. HRMS (ESI^+^): Calcd for C_16_H_25_F_2_N_6_O_13_P_2_ [M+H]^+^: 609.0923; Found: 609.0928.

**Diphosphoribose-2-chloroadenosine (2-Cl-ADPR 10l)**

White foam solid (Et_3_N^+^ salt); yield: 33%.

^1^H NMR (400 MHz, D_2_O) δ 8.34 (d, *J* = 3.6 Hz, 1H), 5.92 (d, *J* = 5.7 Hz, 1H), 5.18 (5.25, d, *J* = 4.1 Hz, 0.4H, 5.12, d, *J* = 1.9 Hz, 0.6H), 4.64 (t, *J* = 5.2 Hz, 1H), 4.44 (t, *J* = 4.2 Hz, 1H), 4.30 (s, 1H), 4.21 (t, *J* = 5.2 Hz, 1H), 4.13 (d, *J* = 3.1 Hz, 2H), 3.99 (m, 4H). ^31^P NMR (162 MHz, D_2_O) δ -10.5 (dt, *J* = 44.6, 20.9 Hz). ^13^C NMR (101 MHz, D_2_O) δ 156.1, 153.8, 150.0, 139.9, 117.4, 101.2, 96.4, 87.0, 81.8, 75.2, 74.4, 70.5, 70.3, 66.4, 65.2. HRMS (ESI^+^): Calcd for C_15_H_23_N_5_O_14_P_2_Cl [M+H]^+^: 594.0405; Found: 594.0406.

**1’’-*O*-Methyl-2’’,3’’-*O*-isopropylidene-5’-methylenediphosphate ribose (CH_2_RDP 6a)**

Light yellow foam solid; yield: 70%.

^1^H NMR (400 MHz, D_2_O) δ 5.01 (s, 1H), 4.85 (d, *J* = 5.9 Hz, 1H), 4.67 (1H, partially hidden under HDO peak), 4.33 (t, *J* = 7.2 Hz, 1H), 3.81 (t, *J* = 6.5 Hz, 2H), 3.29 (s, 3H), 2.05 (td, *J* = 19.8, 2.3 Hz, 2H), 1.41 (s, 3H), 1.28 (s, 3H). ^31^P NMR (162 MHz, D_2_O) δ 19.1 (td, *J* = 20.0, 8.3 Hz), 15.5 (td, *J* = 19.8, 9.4 Hz). ^13^C NMR (101 MHz, D_2_O) δ 113.1, 108.6, 85.3, 85.2, 84.2, 81.1, 64.4, 54. 9, 25.3, 23.7. ESI-MS m/z: 362 [M-H]^-^, 361.

**1’’-*O*-Methyl-2’,3’-*O*-isopropylidene-5’-difluoromethylenediphosphate ribose (CF_2_RDP 6b)**

Light yellow foam solid; yield: 70%.

^1^H NMR (400 MHz, D_2_O) δ 5.26 (s, 1H), 5.11 (d, *J* = 5.9 Hz, 1H), 4.93 (1H, partially hidden under HDO peak), 4.59 (t, *J* = 7.2 Hz, 1H), 4.20 (t, *J* = 6.7 Hz, 2H), 3.55 (s, 3H), 1.67 (s, 3H), 1.53 (s, 3H). ^19^F NMR (376 MHz, D_2_O) δ -117.2 (dd, *J* = 87.6, 73.3 Hz). ^31^P NMR (162 MHz, D_2_O) δ 7.1 (tdt, *J* = 87.4, 51.5, 6.1 Hz), 4.5 (td, *J* = 73.2, 51.6 Hz). ^13^C NMR (101 MHz, D_2_O) δ 113.2, 108.7, 85.4, 85.3, 84.3, 81.1, 66.2, 55.0, 25.4, 23.8. ESI-MS m/z: 398 [M-H]^-^, 397.

**1’’-*O*-Methyl-2’’,2’,3’’,3’-*O*-isopropylidene-5’-methylenediphosphoribose-2-iodoadenosine (7m)**

White foam solid (NH_4_^+^ salt); yield: 30%.

^1^H NMR (400 MHz, D_2_O) δ 8.34 (s, 1H), 6.14 (d, J = 3.1 Hz, 1H), 5.34 (dd, J = 5.9, 3.3 Hz, 1H), 5.15 (dd, J = 5.9, 1.4 Hz, 1H), 4.87 (s, 1H), 4.58 (d, J = 5.7 Hz, 2H), 4.43 (d, J = 5.9 Hz, 1H), 4.18 – 3.91 (m, 3H), 3.75 – 3.52 (m, 2H), 3.21 (s, 3H), 2.04 (t, J = 19.8 Hz, 2H), 1.61 (s, 3H), 1.39 (s, 3H), 1.34 (s, 3H), 1.19 (s, 3H). ^31^P NMR (162 MHz, D_2_O) δ 17.7 (m). ^13^C NMR (101 MHz, D_2_O) δ 155.5, 149.5, 140.0, 119.6, 118.4, 114.9, 112.9, 108.4, 89.7, 85.3, 85.2, 85.0, 84.9, 84.1, 83.9, 81.5, 81.0, 64.3, 54.8, 26.1, 25.2, 24.4, 23.5. HRMS (ESI^+^): Calcd for C_23_H_35_N_5_O_13_P_2_I [M+H]^+^: 778.0751; Found: 778.0750.

**1’’-*O*-Methyl-2’’,2’,3’’,3’-*O*-isopropylidene-5’-difluoromethylenediphosphoribose-2-bromoadenosine (7n)**

White foam solid (NH_4_^+^ salt); yield: 50%.

^1^H NMR (400 MHz, D_2_O) δ 8.29 (s, 1H), 6.08 (d, *J* = 3.3 Hz, 1H), 5.30 (dd, *J* = 6.0, 3.4 Hz, 1H), 5.12 (dd, *J* = 6.0, 2.1 Hz, 1H), 4.86 (s, 1H), 4.55 (dd, *J* = 7.3, 4.1 Hz, 2H), 4.44 (d, *J* = 5.9 Hz, 1H), 4.22 – 4.11 (m, 2H), 4.08 (dd, *J* = 14.4, 7.1 Hz, 1H), 3.93 – 3.66 (m, 2H), 3.20 (s, 3H), 1.59 (s, 3H), 1.38 (s, 3H), 1.31 (s, 3H), 1.16 (s, 3H). ^19^F NMR (376 MHz, D_2_O) δ -118.7 (td, *J* = 83.3, 35.4 Hz). ^31^P NMR (162 MHz, D_2_O) δ 4.1 (m). ^13^C NMR (101 MHz, D_2_O) δ 155.8, 149.7, 144.5, 140.0, 117.7, 115.0, 112.9, 108.5, 89.7, 85.0, 84.9, 84.9, 84.8, 84.1, 83.9, 81.3, 80.9, 66.4, 54.8, 26.2, 25.2, 24.4, 23.5. HRMS (ESI^+^): Calcd for C_23_H_33_F_2_N_5_O_13_P_2_Br [M+H]^+^: 766.0702; Found: 766.0696.

**1’’-*O*-Methyl-2’’,2’,3’’,3’-*O*-isopropylidene-5’-difluoromethylenediphosphoribose-2-iodoadenosine (7o)**

White foam solid (NH_4_^+^ salt); yield: 45%.

^1^H NMR (400 MHz, D_2_O) δ 8.26 (s, 1H), 6.11 (d, *J* = 3.4 Hz, 1H), 5.30 (dd, *J* = 6.0, 3.4 Hz, 1H), 5.12 (dd, *J* = 6.0, 2.1 Hz, 1H), 4.86 (s, 1H), 4.53 (d, *J* = 6.2 Hz, 2H), 4.41 (d, *J* = 5.9 Hz, 1H), 4.25 – 4.10 (m, 2H), 4.06 (t, *J* = 7.3 Hz, 1H), 3.88 – 3.64 (m, 2H), 3.20 (s, 3H), 1.59 (s, 3H), 1.38 (s, 3H), 1.31 (s, 3H), 1.16 (s, 3H). ^19^F NMR (376 MHz, D_2_O) δ -118.7 (td, *J* = 83.6, 43.7 Hz). ^31^P NMR (162 MHz, D_2_O) δ 4.1 (m). ^13^C NMR (101 MHz, D_2_O) δ 155.3, 149.3, 139.7, 119.6, 118.4, 115.0, 112.9, 108.5, 89.6, 85.0, 84.9, 84.8, 84.8, 84.1, 83.9, 81.4, 80.9, 66.2, 54.8, 26.2, 25.2, 24.4, 23.5. HRMS (ESI^+^): Calcd for C_23_H_33_F_2_N_5_O_13_P_2_I [M+H]^+^: 814.0563; Found: 814.0566.

**Methylenediphosphoribose-2-iodoadenosine (2-I-CH_2_ADPR 10m)**

White foam solid (NH_4_^+^ salt); yield: 39%.

^1^H NMR (400 MHz, D_2_O) δ 8.34 (s, 1H), 5.95 (d, *J* = 5.3 Hz, 1H), 5.18 (5.25, d, *J* = 4.0 Hz, 0.4H, 5.11, d, *J* = 2.0 Hz, 0.6H), 4.67 (d, *J* = 5.3 Hz, 1H), 4.46 (t, *J* = 4.5 Hz, 1H), 4.32 – 4.26 (m, 1H), 4.24 (t, *J* = 5.3 Hz, 1H), 4.15 – 4.02 (m, 3H), 4.01 – 3.84 (m, 3H), 2.12 (td, *J* = 19.9, 8.4 Hz, 2H). ^31^P NMR (162 MHz, D_2_O) δ 18.0 (m). ^13^C NMR (101 MHz, D_2_O) δ 155.3, 149.5, 139.6, 119.6, 118.5, 101.2, 96.3, 87.1, 83.9, 82.0, 75.2, 74.3, 70.6, 70.2, 64.4, 63.5. HRMS (ESI^+^): Calcd for C_16_H_25_N_5_O_13_P_2_I [M+H]^+^: 683.9969; Found: 683.9969.

**Difluoromethylenediphosphoribose-2-bromoadenosine (2-Br-CF_2_ADPR 10n)**

White foam solid (NH_4_^+^ salt); yield: 24%.

^1^H NMR (400 MHz, D_2_O) δ 8.27 (s, 1H), 5.88 (d, *J* = 5.3 Hz, 1H), 5.16 (5.23, s, 0.4H, 5.09, s, 0.6H), 4.57 (t, *J* = 5.2 Hz, 1H), 4.44 – 4.34 (m, 1H), 4.23 (d, *J* = 3.1 Hz, 1H), 4.17 (s, 3H), 4.12 – 3.83 (m, 4H,). ^19^F NMR (376 MHz, D_2_O) δ -119.0 (m). ^31^P NMR (162 MHz, D_2_O) δ 4.2 (t, *J* = 82.2 Hz). ^13^C NMR (101 MHz, D_2_O) δ 155.9, 149.9, 144.4, 139.8, 117.9, 101.2, 96.3, 87.1, 83.8, 82.2, 75.1, 74.4, 70.4, 70.1, 66.7, 65.5. HRMS (ESI^+^): Calcd for C_16_H_23_F_2_N_5_O_13_P_2_Br [M+H]^+^: 671.9919; Found: 671.9941.

**1’’-*O*-Methyl-difluoromethylenediphosphoribose-2-bromoadenosine (9a)**

White foam solid (NH_4_^+^ salt); yield: 28%.

^1^H NMR (400 MHz, D_2_O) δ 8.30 (s, 1H), 5.89 (d, *J* = 5.4 Hz, 1H), 4.74 (1H, partially hidden under HDO peak), 4.58 (t, *J* = 5.2 Hz, 1H), 4.43 – 4.34 (m, 1H), 4.24 (d, *J* = 2.9 Hz, 1H), 4.18 (s, 2H), 4.11 (dd, *J* = 6.1, 5.0 Hz, 1H), 4.07 – 3.87 (m, 4H), 3.23 (s, 3H).^19^F NMR (376 MHz, D_2_O) δ -118.7 (t, *J* = 82.4 Hz). ^31^P NMR (162 MHz, D_2_O) δ 4.2 (m). ^13^C NMR (101 MHz, D_2_O) δ 155.9, 149.9, 144.4, 139.7, 117.9, 107.8, 90.2, 87.1, 83.9, 81.5, 74.4, 74.0, 70.7, 70.1, 67.0, 65.5, 55.1. HRMS (ESI^+^): Calcd for C_17_H_25_F_2_N_5_O_13_P_2_Br [M+H]^+^: 686.0076; Found: 686.0051.

**1’’-*O*-Methyl-2’’,3’’-*O*-isopropylidene-5’-difluoromethylenediphosphoribose-2-bromoadenosine (8d)**

White foam solid (NH_4_^+^ salt); yield: 33%.

^1^H NMR (400 MHz, D_2_O) δ 8.37 (s, 1H), 5.94 (d, *J* = 5.6 Hz, 1H), 4.88 (s, 1H), 4.69 – 4.61 (t, *J* = 5.2 Hz, 1H), 4.57 (d, *J* = 6.0 Hz, 1H), 4.47 (d, *J* = 5.9 Hz, 1H), 4.42 (dd, *J* = 5.0, 3.8 Hz, 1H), 4.28 (d, *J* = 1.8 Hz, 1H), 4.22 (d, *J* = 4.5 Hz, 2H), 4.11 (t, *J* = 7.3 Hz, 1H), 3.90 – 3.73 (m, 2H), 3.21 (s, 3H), 1.29 (s, 3H), 1.14 (s, 3H). ^19^F NMR (376 MHz, D_2_O) δ -118.6 (t, *J* = 83.5 Hz). ^31^P NMR (162 MHz, D_2_O) δ 4.2 (m). ^13^C NMR (101 MHz, D_2_O) δ 156.1, 150.2, 144.5, 139.8, 118.0, 112.9, 108.5, 87.0, 84.8, 84.8, 84.2, 84.1, 80.9, 74.4, 70.4, 66.2, 65.6, 54.8, 25.1, 23.4. HRMS (ESI^+^): Calcd for C_20_H_29_F_2_N_5_O_13_P_2_Br [M+H]^+^: 726.0389; Found: 726.0404.

**Difluoromethylenediphosphoribose-2-iodoadenosine (2-I-CF_2_ADPR 10o)**

White foam solid (NH_4_^+^ salt); yield: 25%.

^1^H NMR (400 MHz, D_2_O) δ 8.25 (s, 1H), 5.90 (d, *J* = 5.3 Hz, 1H), 5.12 (5.23, s, 0.4H, 5.09, s, 0.6H), 4.57 (t, *J* = 5.2 Hz, 1H), 4.42 – 4.37 (m, 1H), 4.24 (d, *J* = 3.1 Hz, 1H), 4.18 (s, 2H), 4.11 – 3.81 (m, 5H). ^19^F NMR (376 MHz, D_2_O) δ -118. 9 (m). ^31^P NMR (162 MHz, D_2_O) δ 4.2 (t, *J* = 82.3 Hz). ^13^C NMR (101 MHz, D_2_O) δ 155.3, 149.4, 139.4, 119.5, 118.4, 101.1, 96.3, 87.1, 83.8, 81.5, 75.1, 74.4, 70.4, 70.1, 66.7, 65.5. HRMS (ESI^+^): Calcd for C_16_H_23_F_2_N_5_O_13_P_2_I [M+H]^+^: 719.9780; Found: 719.9762.

**1’’-*O*-Methyl-difluoromethylenediphosphoribose-2-iodoadenosine (9b)**

White foam solid (NH_4_^+^ salt); yield: 30%.

^1^H NMR (400 MHz, D_2_O) δ 8.21 (s, 1H), 5.87 (d, *J* = 5.1 Hz, 1H), 4.70 (1H, hidden under HDO peak), 4.54 (t, *J* = 5.1 Hz, 1H), 4.42 – 4.32 (m, 1H), 4.21 (d, *J* = 3.3 Hz, 1H), 4.19 – 4.05 (m, 3H), 4.05 – 3.84 (m, 4H), 3.20 (s, 3H). ^19^F NMR (376 MHz, D_2_O) δ -118.8 (t, *J* = 82.2 Hz). ^31^P NMR (162 MHz, D_2_O) δ 4.1 (td, *J* = 82.2, 12.0 Hz). ^13^C NMR (101 MHz, D_2_O) δ 155.2, 149.3, 139.4, 119.5, 118.4, 107.8, 105.0, 87.2, 83.7, 81.4, 74.4, 74.0, 70.7, 70.0, 67.0, 65.4, 55.1. HRMS (ESI^+^): Calcd for C_17_H_25_F_2_N_5_O_13_P_2_I [M+H]^+^: 733.9937; Found: 733.9949.

**1’’-*O*-Methyl-2’’,3’’-*O*-isopropylidene-5’-difluoromethylenediphosphoribose-2-iodoadenosine (8e)**

White foam solid (NH_4_^+^ salt); yield: 29%.

^1^H NMR (400 MHz, D_2_O) δ 8.27 (s, 1H), 5.91 (d, *J* = 5.4 Hz, 1H), 4.84 (s, 1H), 4.61 (t, *J* = 5.2 Hz, 1H), 4.52 (d, *J* = 6.0 Hz, 1H), 4.44 – 4.36 (m, 2H), 4.25 (d, *J* = 2.2 Hz, 1H), 4.21 – 4.18 (m, 2H), 4.08 (t, *J* = 7.3 Hz, 1H), 3.86 – 3.69 (m, 2H), 3.18 (s, 3H, CH_3_), 1.26 (s, 3H), 1.11 (s, 3H). ^19^F NMR (376 MHz, D_2_O) δ -118.7 (t, *J* = 83.4 Hz). ^31^P NMR (162 MHz, D_2_O) δ 4.12 (m). ^13^C NMR (101 MHz, D_2_O) δ 155.3, 149.6, 139.4, 119.5, 118.5, 112.9, 108.5, 87.1, 84.8, 84.7, 84.1, 83.9, 80.8, 74.4, 70.3, 66.3, 65.6, 54.8, 25.1, 23.5. HRMS (ESI^+^): Calcd for C_20_H_29_F_2_N_5_O_13_P_2_I [M+H]^+^: 774.0250; Found: 774.0237.
